# Biotic and Climatic Velocity Identify Contrasting Areas of Vulnerability to Climate Change

**DOI:** 10.1371/journal.pone.0140486

**Published:** 2015-10-14

**Authors:** Carlos Carroll, Joshua J. Lawler, David R. Roberts, Andreas Hamann

**Affiliations:** 1 Klamath Center for Conservation Research, Orleans, CA 95556, United States of America; 2 School of Environmental and Forest Sciences, University of Washington, Seattle, WA, 98195, United States of America; 3 Department of Renewable Resources, University of Alberta, Edmonton, AB, T6G2H1, Canada; 4 Department of Biometry and Environmental System Analysis, University of Freiburg, 79106, Freiburg, Germany; University of Sydney, AUSTRALIA

## Abstract

Metrics that synthesize the complex effects of climate change are essential tools for mapping future threats to biodiversity and predicting which species are likely to adapt in place to new climatic conditions, disperse and establish in areas with newly suitable climate, or face the prospect of extirpation. The most commonly used of such metrics is the velocity of climate change, which estimates the speed at which species must migrate over the earth’s surface to maintain constant climatic conditions. However, “analog-based” velocities, which represent the actual distance to where analogous climates will be found in the future, may provide contrasting results to the more common form of velocity based on local climate gradients. Additionally, whereas climatic velocity reflects the exposure of organisms to climate change, resultant biotic effects are dependent on the sensitivity of individual species as reflected in part by their climatic niche width. This has motivated development of biotic velocity, a metric which uses data on projected species range shifts to estimate the velocity at which species must move to track their climatic niche. We calculated climatic and biotic velocity for the Western Hemisphere for 1961–2100, and applied the results to example ecological and conservation planning questions, to demonstrate the potential of such analog-based metrics to provide information on broad-scale patterns of exposure and sensitivity. Geographic patterns of biotic velocity for 2954 species of birds, mammals, and amphibians differed from climatic velocity in north temperate and boreal regions. However, both biotic and climatic velocities were greatest at low latitudes, implying that threats to equatorial species arise from both the future magnitude of climatic velocities and the narrow climatic tolerances of species in these regions, which currently experience low seasonal and interannual climatic variability. Biotic and climatic velocity, by approximating lower and upper bounds on migration rates, can inform conservation of species and locally-adapted populations, respectively, and in combination with backward velocity, a function of distance to a source of colonizers adapted to a site’s future climate, can facilitate conservation of diversity at multiple scales in the face of climate change.

## Introduction

Metrics that synthesize the complex effects of climate change are essential tools for mapping future threats to biodiversity. As climate shifts over the coming decades, such metrics can help us predict which species are likely to adapt in place to new climatic conditions, disperse and establish in areas with newly suitable climate, or face the prospect of extirpation [1]. Such information also allows us to evaluate whether specific sites, such as existing protected areas, will serve as refugia or face loss of species and consequent changes in ecosystem processes [2].

**Fig 1:**
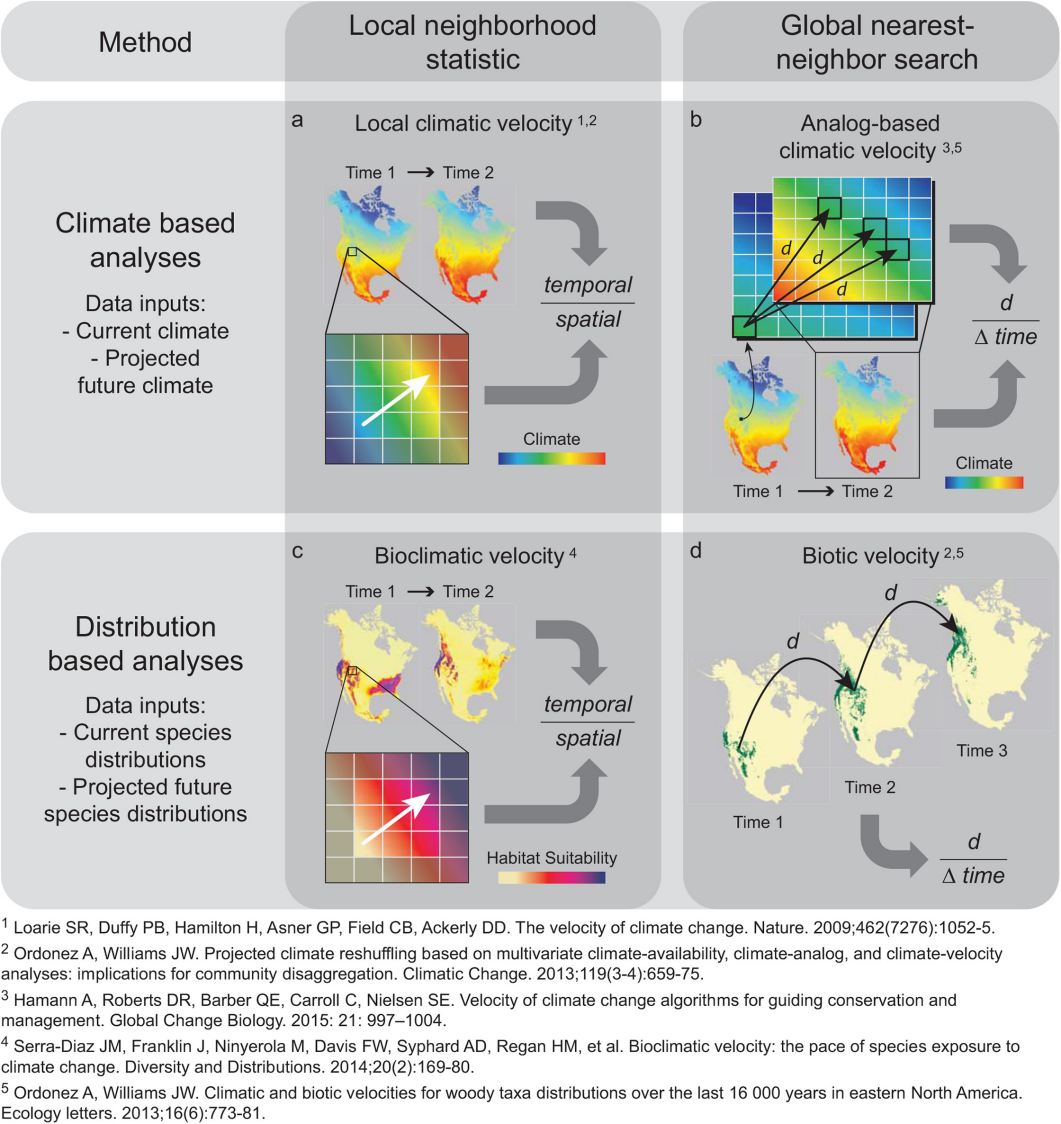
Conceptual diagram contrasting four types of velocity metric used in climate adaptation planning, categorized in terms of the method and type of data used.

The most commonly used of such metrics is the velocity of climate change, which estimates the speed at which species must migrate over the earth’s surface to maintain constant climatic conditions. As originally proposed, climatic velocity is derived by dividing the temporal rate of projected climate change by the current gradient of climate variability across a spatial neighborhood [3]. This method, which has been termed “local” velocity [4] (Fig 1A), only considers spatial variability within the immediate neighborhood of a location. Consequently, the rate of migration necessary to maintain constant climate conditions may be underestimated in alpine and arctic regions if velocity vectors point toward climatic cul-de-sacs, i.e. by pointing beyond mountain tops or polar edges of continents [5, 6]. Secondly, velocity may approach infinity in flat areas, even if similar future climates are found just beyond the focal neighborhood.

Because of these factors, a metric based on the actual distance between a location and the location where a similar (analogous) climate will be found in the future forms a valuable complement to local velocity [4, 7, 8]. Deriving such “analog-based” velocity (Fig 1B) can be computationally challenging, because it requires a search over the entire extent of the data to identify the most spatially-proximate cell which is an analog of the focal cell based on the environmental attribute(s) of interest. However, recent advances in efficient nearest-neighbor search algorithms have made such global searches computationally feasible for high-resolution raster datasets [9]. The shift of the search radius from a neighborhood to the entire extent of the data enhances the relevance of the velocity metric, especially for mobile species that may disperse over larger distances than can be evaluated in a neighborhood-based search.

**Fig 2:**
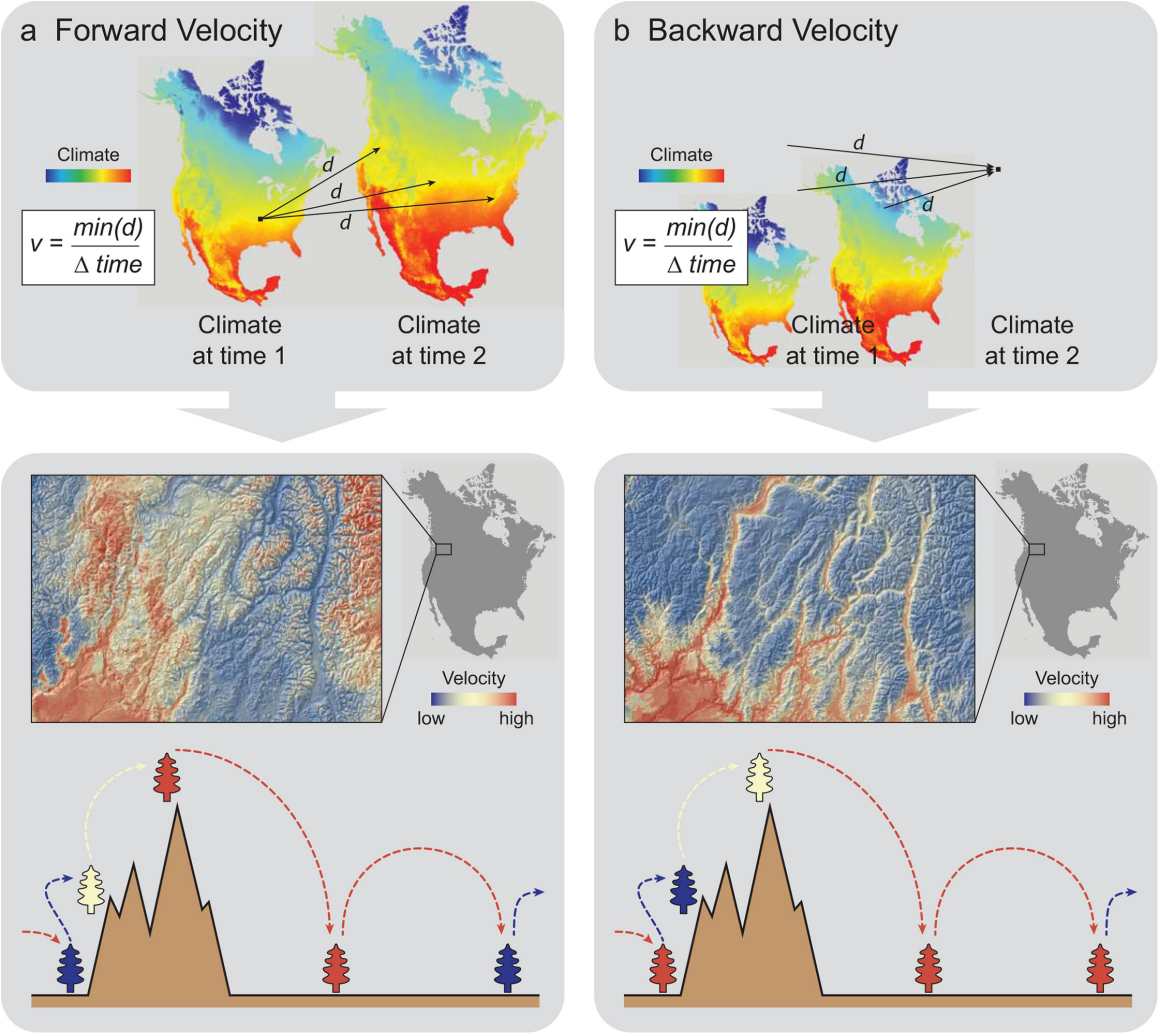
Conceptual diagram contrasting a) forward and b) backward velocity. Upper panels illustrate contrasts between how the two metrics are derived. Lower panels show a high resolution map of the contrast between forward and backward velocity in mountainous terrain, as well as a diagram illustrating the source of these contrasts. The colors of trees in the diagram correspond to the velocity expected in that topographic position, with the corresponding arrows of the same color either originating from (forward velocity) or terminating at that topographic position.

Additionally, nearest-neighbor search allows calculation of both forward velocities (the distance from current climate locations to the nearest site with an analogous future climate; Fig 2A) and backward velocities (the distance from projected future climate cells back to analogous current climate locations; Fig 2B). At first glance, these two forms of velocity based on the same inputs appear to differ only in their opposing time directions. However, the two calculations can yield profoundly different results which provide contrasting but complementary information to support conservation of species and their populations. Forward velocity reflects the minimum distance an organism in the current landscape must migrate to maintain constant climate condition. Because forward velocity reflects the exposure of organisms to climate change, the metric is particularly relevant in assessing the conservation status of species and their populations under climate change. Forward velocity will often be high in alpine areas because reaching the nearest analogous future climate may require dispersal to distant higher elevation mountaintops (Fig 2A, lower panel).

Backward velocity reflects the minimum distance, given the projected future conditions at a site, that a climatically-adapted organism would have to migrate to colonize the site. Backward velocity reflects the relative difficulty of species faces in colonizing newly suitable habitat via dispersal or gene flow (e.g., via pollen) [7]. Backward velocity, unlike forward velocity, is generally low in alpine areas, because adapted organisms can reach the site from nearby downslope locations. Conversely, valley bottom habitat may lack nearby climate analogs, and organisms would have to travel longer distances to colonize these locally new habitat conditions (Fig 2B, lower panel).

Forward velocity measures the distance from a single source to multiple future destinations (Fig 2A, top panel). Conversely, backward velocity considers the distance between disparate source locations and a single future destination (Fig 2B, top panel). Therefore, the use of these two metrics in planning will typically imply a contrasting focus on species/populations (forward velocity) or sites (backward velocity). While species inhabiting a site with high forward velocity are potentially threatened with extinction, a site with high backward velocity is threatened with holding a depauperate complement of species adapted to its future climate, with consequent effects on ecosystem function and services.

The distinction between local and analog-based velocity is also relevant for velocities based on projections from climatic niche models (a type of model based on correlations between species distributions and current climatic conditions [10]). A local metric, termed “bioclimatic” velocity [11], can be calculated based on the modeled probability of species occurrence (inferring habitat suitability). This habitat suitability metric is treated as equivalent to a climate variable in local climatic velocity, in that velocity is derived by dividing the rate of change over time in a continuous habitat suitability metric by the rate of change in that metric within the local neighborhood (Fig 1C). In contrast, an analog-based metric, termed “biotic velocity” [4, 12], represents the distance between a site and the nearest site projected to be climatically suitable for the species under future projected climates (Fig 1D). The metric can be reported on a per-species basis or averaged across a taxa group. Because it is based on a binary classification of the landscape into suitable and unsuitable areas, biotic velocity sacrifices information on local habitat gradients in order to incorporate information on broad-scale patterns in range shifts.

Several studies have determined that historical climatic velocity is correlated with past species range shifts [13–15]. In contrast to historical studies, forward-looking comparisons rely on projected range shifts as a response variable, which imperfectly represents the actual pattern of future species movements. However, comparison of biotic and climatic velocity based on future climate projections is critical in informing planners as to whether the higher level of model complexity represented by climatic niche models results in qualitatively different threat patterns and conservation priorities. For example, a strategy for identification and conservation of refugia [16] would be more complex if areas of low biotic velocity did not overlap with areas of low climatic velocity. In general, consideration of a suite of climate-related metrics results in more robust threat assessments than could a focus on any one measure in isolation [1, 17].

Climate displacements derived from multiple bioclimatic variables show more complex and informative patterns than those shown by the magnitude of temperature change alone [18–20]. We developed multivariate climatic velocity for North and South America based on a principal components analysis of 37 bioclimatic variables, using data derived from ten GCMs for the SRES A2 emissions scenario [21] (S1 Table). We developed biotic velocity from multivariate climatic niche models for 2954 terrestrial species (1818 birds, 723 mammals and 413 amphibians) based on a previously-published analysis that projected shifts in the geographic distribution of climatic suitability from a current historical (1961–1990) to a future (2071–2100) habitat time period, based on the ten GCM projections [22]. The hemispheric extent allowed us to compare the two metrics across a diverse set of biomes that vary widely in their climatic and biogeographic attributes. We also compared the distribution of disappearing climates and species.

Previous studies have mapped local or neighborhood-based climatic velocity [3] and disappearing climates [23] at a global scale, as well as distance to future analogous climates in Europe [24]. Analog-based biotic velocity has been assessed between current and paleoclimates for North American trees [12], and between current and future projected climates for Western Hemisphere mammals [25]. Local bioclimatic velocity has been analyzed for California tree species [11]. In this study, we provide the first comparison of analog-based climatic and biotic velocity between current and projected future climate over a broad taxonomic and geographic scope.

Because analog-based velocity searches beyond a local neighborhood (Fig 1), the metric effectively integrates factors that influence climate at a range of spatial scales, including local topographic gradients, regional topographic position, and location in relationship to continental margins and synoptic weather systems. In this study, we use two examples to demonstrate how such information on broad-scale patterns of exposure and sensitivity enhances the relevance of the velocity metric to conservation planning. Firstly, we use analog-based velocity to evaluate how climatic vulnerability varies with latitude. We ask whether potential threats are greater in equatorial, temperate, or boreal regions, and whether these threats derive primarily from the magnitude of climate change or the differential sensitivity of taxa in different regions. Secondly, we demonstrate how to integrate results from a suite of complementary velocity metrics, using an example focusing on climate-related threats to protected areas of the Western Hemisphere. By placing the sometimes confusing variety of previously-proposed velocity metrics in a coherent framework, and demonstrating how they can be integrated to inform a multi-faceted threat assessment, we hope to stimulate their more widespread use in the increasing number of planning efforts focused on conserving landscape-scale climate resilience and adaptation potential.

## Materials and Methods

### Climatic velocity

Climate data were downscaled to a 50km by 50km grid using locally weighted, lapse-rate-adjusted interpolation. Monthly climate data from 1961 to 1990 were then averaged to produce a base-line time period. Future climate projections were taken from ten general circulation model (GCM) simulations archived by the World Climate Research Program Coupled Model Intercomparison Project phase 3 (CMIP3)(S1 Table). Climate projections were averaged over a 30-year period from 2071 to 2100 and represent climates simulated for a mid-to-high (SRES A2) greenhouse-gas emissions scenario [21]. Thirty-seven bioclimatic variables were developed from the primary climate data [22] (S2 Table).

A mid-to-high emissions scenario (SRES A2) was selected because the recent global CO_2_ emissions trajectory has fallen at the high end of the range of projections used in IPCC scenarios [26]. Use of previously-published niche models limited the analysis to CMIP3 data rather than the recently-released CMIP5 projections. However, because CMIP5 projections are an incremental advance over CMIP3 data [27, 28], this decision does not substantially limit the relevance of our results as a tool for threat assessment. Temperature projections from the SRES A2 scenario are intermediate to those of CMIP5 representative concentration pathways (RCP) 6.0 and 8.5 [28, 29].

We used a direct estimate of climatic velocity, or the distance between a location and the nearest location with analogous climate in the future [7]. Climate analogs were identified using multivariate Euclidean climate distance estimated from the first 5 axes of a principal components analysis of the 37 bioclimatic variables. Use of multivariate Euclidean distance based on PCA scores reduced the effect of collinearity between bioclimatic variables and approximated a Mahalanobis distance based on the original set of variables.

Principal components analysis of the 37 bioclimatic variables resulted in first and second PCA component dominated by temperature and precipitation, respectively. These two components captured 57.2% and 20.6% of total variance, respectively. The five components capturing greater than 2% of variance each, which cumulatively captured 91.4% of total variance, were used in the subsequent velocity calculations. We used equally spaced intervals to divide current and future climate space into multivariate bins. Because the width of each bin was maintained across all 5 PCA axes, the number of bins on each axis, and its importance in determining a climate analog, is approximately proportional to the variance explained by that axis.

The degree of similarity which constitutes an analogous climate type (i.e., the width of a climate bin) is a key parameter in derivation of analog-based velocity. In a two-dimensional plot of the first two PCA components, these bins can be visualized as rectangular areas with similar climate values in multivariate space. Given enough precision in measurements, no two grid cells will have the same climate value. As bin width widens and precision decreases, velocity values decrease to the point where information is lost because many areas show zero velocity (S1A Fig)[7]. Conversely, as bin width narrows past a certain threshold, the proportion of no-analog climates, which also hold limited information value, sharply increases (S1B Fig) [7]. We found that a bin width of 2 PCA units optimized the information value of the resulting velocity maps by balancing loss of information due to excessively broad bins versus domination of the results by disappearing or no-analog climates due to excessively narrow bins (S1 Fig).

For each unique climate type (bin), we identified cells (sites or pixels) within that type under both current and projected climates. Then, we used fast approximate-nearest-neighbor algorithms (using the R package yaImpute [30]; see S1 File and http://adaptwest.databasin.org for code) to identify, for each current cell, the nearest cell of the same type under future climates. This distance provided the basis for deriving forward climatic velocity. Additionally, for each future cell, we identified the nearest neighboring cell of the same type under current climate. This distance provided the basis for deriving backward climatic velocity [7].

Cells for which no match can be found in the other time period represent cells which have velocities that extend beyond the analysis region. Although these areas could be assigned an arbitrarily high velocity value, this would obscure patterns in the velocity results. Therefore, the probability of disappearance of climate types (as well as of species; see below) was tabulated separately from calculations of climatic velocity. Disappearing or no-analog climates can also arise as an artifact of the arbitrary boundaries between climate bins. We incrementally offset bin boundaries over 100 replicated velocity calculations and averaged the results to eliminate this effect. We then averaged velocities from the ten GCMs to derive a mean velocity value.

### Biotic velocity

Climatic niche models for the North and South American continents, at 50 km resolution, were drawn from a previously-published study [22], which assessed the potential effects of climate change on distribution of 2954 vertebrate species (1818 birds, 723 mammals, and 413 amphibians) in the Western Hemisphere. Species distribution data were taken from digital range maps for birds [31], mammals [32] and amphibians (data available online, www.globalamphibians.org). For birds, only breeding ranges were included. Range maps are scale-dependent abstractions of species distributions which overestimate species’ occurrences [33]. However, they are useful in taxonomically comprehensive assessments where point locations of species’ occurrences are unavailable for a subset of the species considered [34, 35]. The 50 km resolution was chosen to strike a balance between inaccuracies due to applying a fine-resolution grid to relatively coarse resolution digital-range maps and inaccuracies due to mapping climate at too coarse a resolution. Niche models were developed using the same 37 bioclimatic variables used in deriving climatic velocity (S2 Table).

Niche models were built using random forest predictors, an ensemble-based machine-learning approach based on combining predictions from multiple classification or regression trees built using random subsets of the data and predictor variables [36]. For each model, a threshold for predicting presence was determined using the receiver-operating characteristic curve with the assumption that correctly predicting a presence was as important as predicting an absence [37]. Models for 2954 species were able to correctly predict at least 90% of the absences and 80% of the presences in the reserved test data sets. These models were then used to project areas that will likely be climatically suitable under the ten GCM projections of future climates.

We calculated biotic velocity based on climatic niche model projections from each of the ten GCMs. To calculate forward velocity, we assigned each cell within the current range of the species a velocity based on the distance to the nearest cell of future climatically suitable habitat (see S1 File and http://adaptwest.databasin.org for code). To calculate backward velocity, we assigned each cell within the future projected range of a species a velocity based on the distance to the nearest cell of current climatically suitable habitat. We averaged resultant velocities to derive a mean value for each of the three taxa groups (birds, mammals, and amphibians). For each cell, we also calculated the probability of a species disappearing at that site. We used probability rather than number of species disappearing at each site to reduce correlation of the metric with gradients in current species diversity.

### Comparison of climatic and biotic velocity

We compared climatic and biotic velocity metrics using four methods. Firstly, we calculated the Spearman rank correlation coefficients between the metrics. Secondly, we used generalized-additive models [38] to assess latitudinal gradients in climatic and biotic velocity. We assessed whether the relative intensity of climatic velocity was greatest in equatorial, temperate, or boreal regions, and whether biotic velocity mirrored or contrasted the latitudinal patterns shown by climatic velocity. If climatic and biotic velocity values peak in different latitudinal regions, this would imply that relative importance of exposure vs. sensitivity in producing climate-related vulnerability differs with latitude. Specifically, we hypothesized that climatic velocity would peak in boreal regions, which have experienced the greatest magnitude of anthropogenic climate change to date [39], but that the generally narrower ranges of tropical species would cause the peak values of biotic velocity to occur outside the polar regions.

Thirdly, we compared the distribution of velocity values between latitudinal zones and between major climatic zones (defined in terms of the Koppen-Gieger system [40]) using plots based on kernel density estimators and beanplots [41]. We evaluated three distinct types of variation in the results. On a per-cell basis, we calculated variation (standard deviation) in velocity values between GCMs and between species within a taxa group. Taking the mean biotic velocities for each cell, we evaluated their variation across space to illustrate the degree of contrast between refugia and high velocity areas within a climate zone or latitudinal band. Taking the mean velocities for each species within a climatic zone, we evaluated variation between species within a taxa group to illustrate whether geographic patterns were general to most species or driven by a subset of highly vulnerable species.

Lastly, we analyzed the vulnerability of major protected areas (i.e., those of area >200 km^2^; data available from www.protectedplanet.net/) of the Americas based on the various velocity metrics, by calculating the mean of velocity values within the extent of the protected area. Plotting the relative ranking of protected areas based on the four metrics (forward and backward climatic and biotic velocity) demonstrated how multiple metrics may be visually integrated and interpreted to support a multi-faceted threat assessment.

## Results

Mean forward and backward climatic velocities (3.58 and 3.52 km/year (SD 2.71 and 2.55 km/year) respectively) were an order of magnitude greater than mean forward and backward biotic velocities (0.433 and 0.480 km/year (SD 0.382 and 0.782 km/year), respectively for all species combined). Whereas mean forward biotic velocity for amphibian species (0.698 km/year (SD 0.710 km/year)) was greater than that for birds and mammals (0.427 and 0.343 km/year, (SD 0.392 and 0.320 km/year) respectively), the reverse was true of backward biotic velocity (0.170, 0.546, 0.495 km/year (SD 0.218, 0.961, and 0.764 km/year) for amphibian, bird, and mammal species, respectively). These standard deviations represent variation across space in mean velocities calculated on a per-cell basis.

When the standard deviation of species-specific velocities was calculated for each cell and averaged across space, mean values were 0.877 and 1.55 km/year, respectively, for forward and backward biotic velocities. The standard deviation between mean biotic velocities for different GCMs was lower at 0.323 and 0.248 km/year, respectively, for forward and backward biotic velocities.

**Fig 3:**
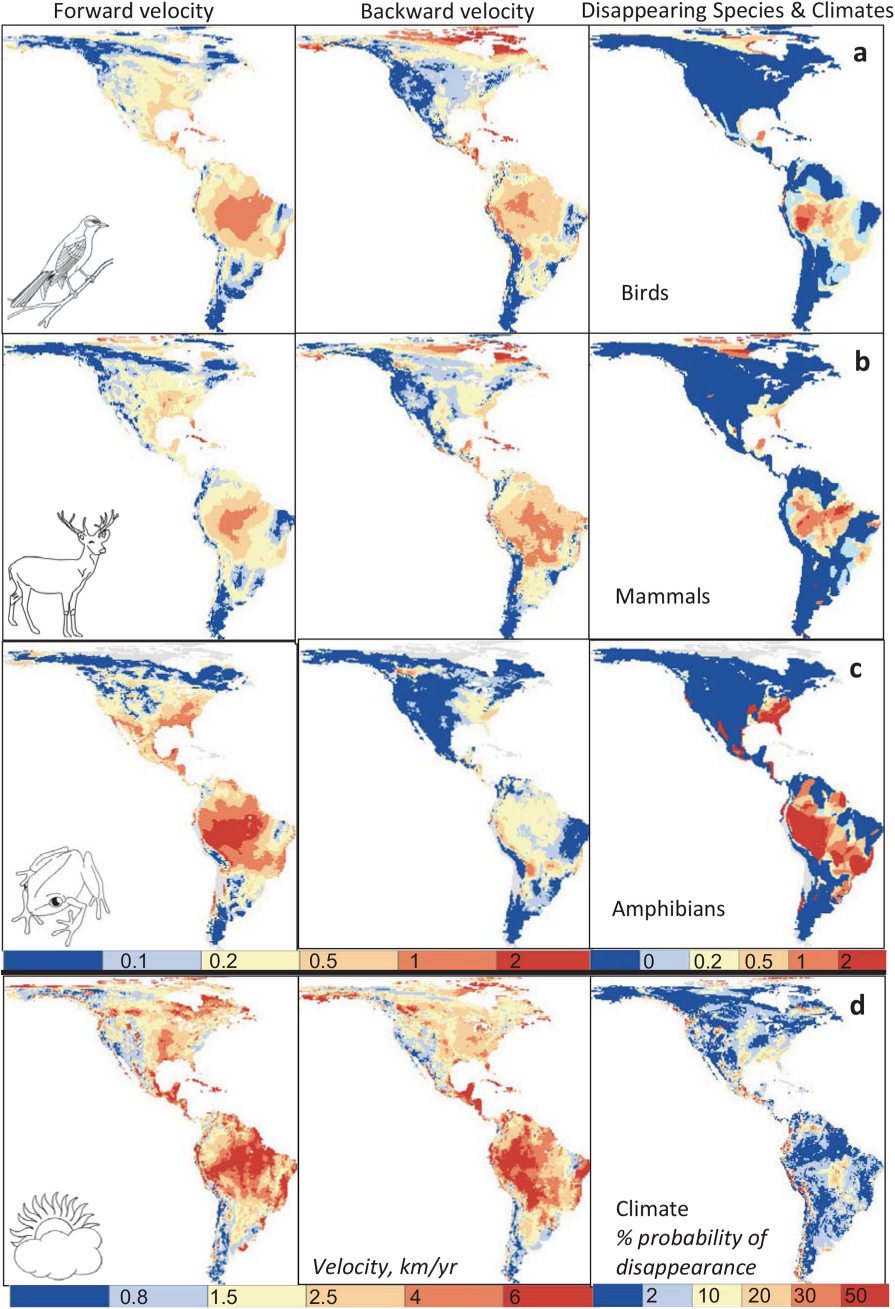
Geographic patterns of mean biotic (a-c) and climatic (d) velocity, and disappearing species and climates in the Western Hemisphere. Results are shown for the period 1961–2100 under the SRES A2 scenario. Note the different scales used for biotic and climatic velocity.

The geographic distribution of high velocity areas differed between taxa and between biotic and climatic velocity (Fig 3). Variation in biotic velocity between species within taxa groups (birds, mammals, amphibians) and between GCMs was greatest in the Amazon Basin, Central America, the southeastern US, and Arctic regions (S2 Fig).

Distribution plots of variation across space (within climatic zones or latitudinal bands) in mean velocity values for taxa groups showed that hotspots of low and high velocity (as indicated by skewed or multimodal distributions) exist, especially at higher latitudes (S3 and S4 Figs). Variation across taxa in species-specific mean velocities within geographic zones indicated that taxa inhabiting arid, cold, and polar zones were more diverse in their levels of relative vulnerability than were equatorial or temperate taxa (S5 Fig).

High climatic velocities were located in both flat and upper montane areas (where climate types are “pushed off” mountaintops), as well as in peninsulas and islands. While backward biotic velocities follow generally similar patterns, the distribution of forward biotic velocities diverged from these patterns, suggesting greater influence from biogeographic factors. Correlation between forward climatic and biotic velocity was thus lower than between backward velocities (Spearman rank correlation *r* ^s^ = 0.523 vs. 0.619 for all species). Disappearing climates were poorly correlated with (*r* ^s^ = 0.184) and were more broadly distributed than areas of disappearing species, and were more prevalent in montane areas.

**Fig 4:**
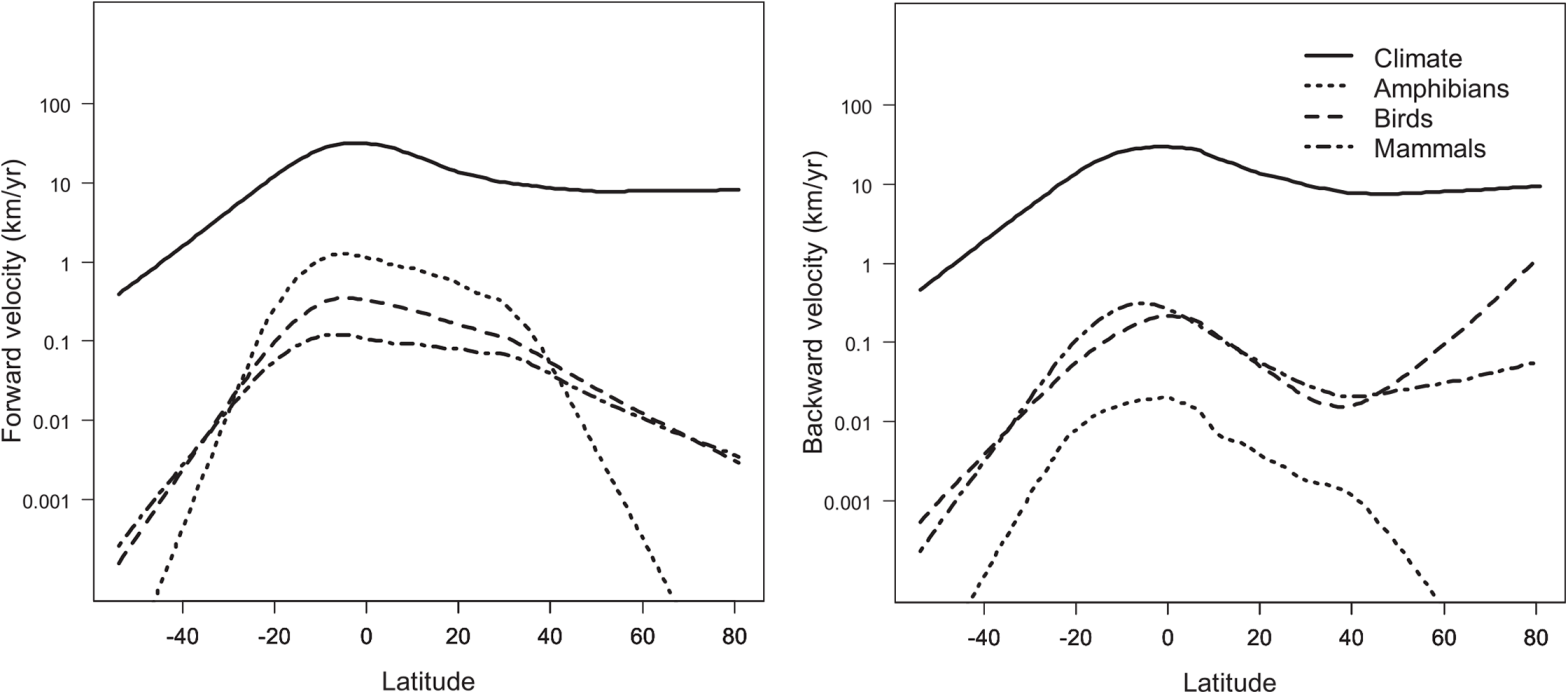
Latitudinal gradients in (a) forward and (b) backward climatic and biotic velocity. Curves were fit using generalized additive models to a dataset of grid cells covering North and South America at a resolution of 50km (*n* = 15,318).

**Fig 5:**
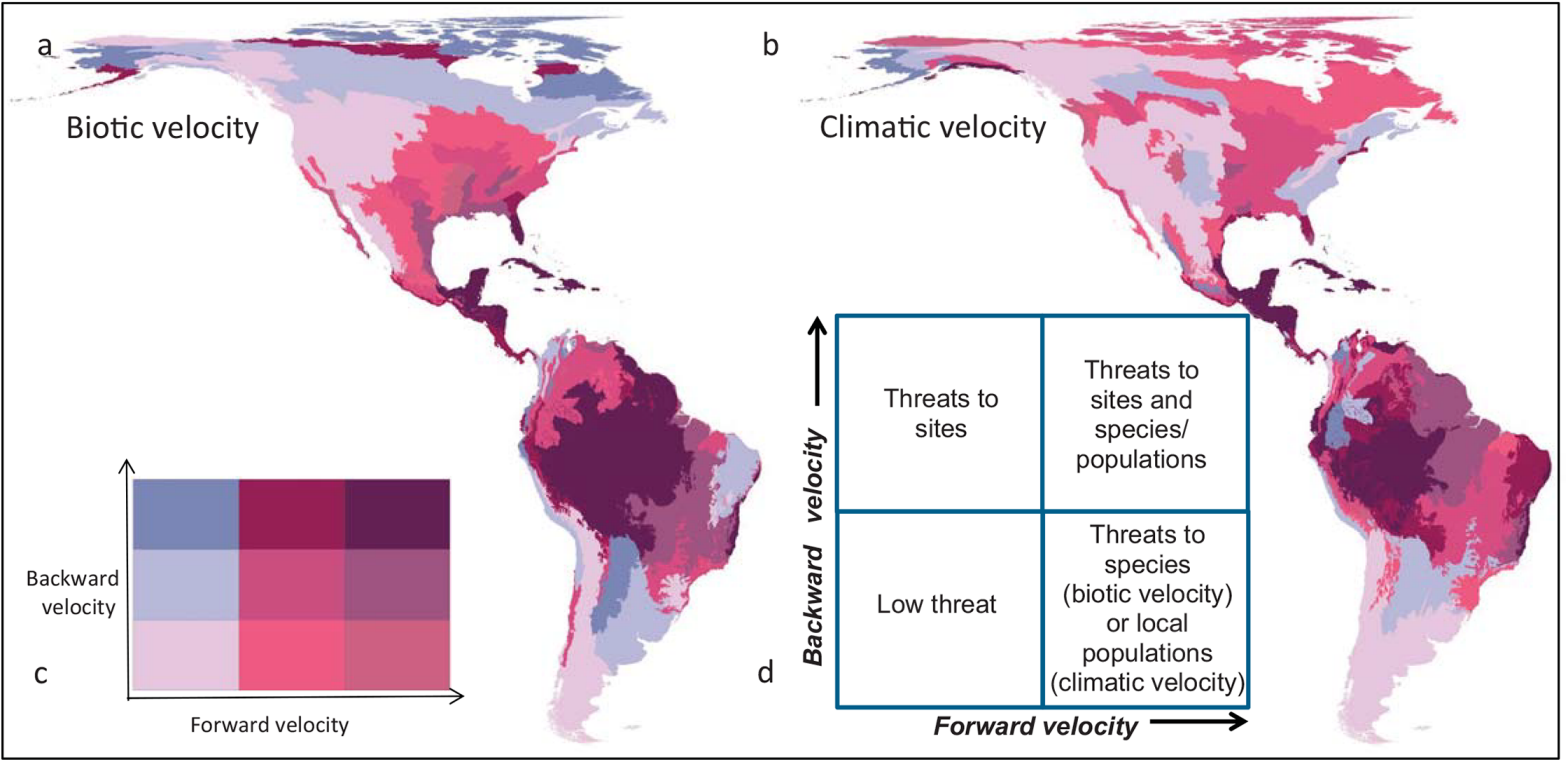
Magnitude of (a) biotic and (b) climatic velocity for Western Hemisphere ecoregions. Results are shown for the period 1961–2100 under the SRES A2 scenario. The bivariate chloropleth plot jointly represents both forward and backward velocity, allowing categorization of ecoregions into the 4 threat quadrants shown in the inset (d). To create the 9 categories shown in (c), log-transformed velocity values (km/year) were grouped into three equal area quantiles along each axis.

Correlation between biotic and climatic velocity was greatest in equatorial regions (S5 Fig). Both biotic and climatic velocities were greatest at equatorial latitudes (Fig 4 and S3 Fig). Backward biotic velocity was high in both equatorial and polar regions. Forward and backward climatic velocity showed a similar pattern combining a peak in equatorial regions with moderately high velocities in north temperate and polar regions (Fig 4). Ecoregions showing high levels of both forward and backward velocity were concentrated in the northern Amazon Basin and Central America (Fig 5).

**Fig 6:**
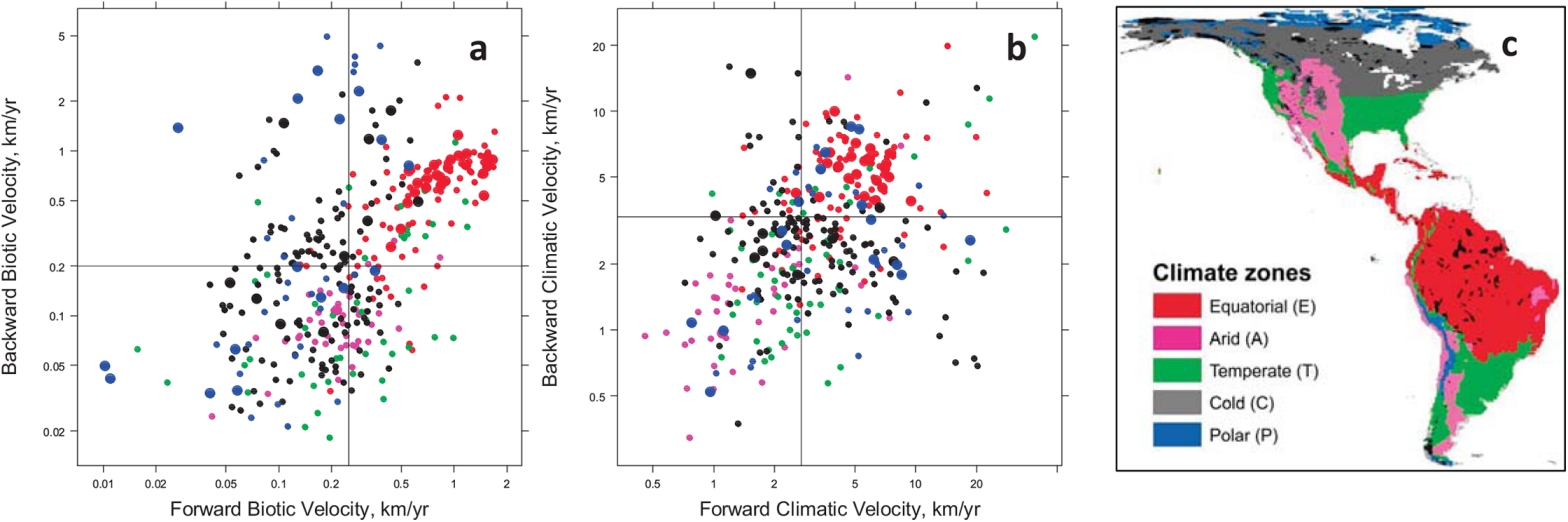
Velocity-based vulnerability assessment of major protected areas of the Americas based on biotic (a) and climatic (b) velocity. Symbol colors are based on colors of major climatic zones in (c). All strictly protected areas (categories I and II) greater than 200km^2^ in area are shown (*n* = 362) with areas larger than 10,000 km^2^ (*n* = 51) shown as larger symbols. Axes were placed at median x and y values to illustrate application of the four-quadrant framework shown in Fig 5D.

The vulnerability of major protected areas also differed between climatic regions (Fig 6). Equatorial protected areas were highly vulnerable from the perspective of both forward and backward biotic velocities as well as forward climatic velocity. North temperate protected areas showed greater threats from forward than backward velocity. Arctic (polar) protected areas showed a wide range of vulnerability to both climatic velocity and forward biotic velocity.

## Discussion

Biotic responses to climate change can be conceptualized as lying along three axes: space, time, and ecology [42]. The simplest metrics for assessing vulnerability to climate change consider only variation across space (e.g., diversity of elevation, aspect, or current climate)[43, 44]. Climatic velocity additionally considers variation along a temporal axis. Biotic velocity adds consideration of a third ecological axis by incorporating information on the climatic niche breadth of individual species.

When seen in this framework, the velocity metrics considered here provide complementary sources of information for threat assessment and conservation planning under climate change. To provide informative results, the analog-based climatic velocity method must use a relatively narrow range of climatic conditions to define what constitutes a “match” between current and projected future climates. Climatic velocities therefore represent an upper bound of migration requirements because they effectively assume negligible climatic tolerances or adaptive capacities of populations inhabiting a site.

In contrast, biotic velocity is derived from climatic niche models which assume climatic tolerances based on a species’ realized niche. Biotic velocities provide a lower estimate of migration requirements which assumes local populations can adapt to any climatic conditions encountered within the full range of the species distribution. If a species’ fundamental niche is broader than the realized niche evident from current distribution data, or if adaptation allows a species to broaden its climatic niche over time, biotic velocity estimates will still be greater than the actual movement rate required for species persistence.

The large contrast between our mean estimates of climatic and biotic velocity demonstrates the contrast in magnitude that might be expected between estimates of the climatic tolerances of a species as a whole and estimates that assume a population is narrowly adapted to local climatic conditions. The magnitude of analog-based climatic velocity does depend in part on the parameter that defines how similar two climates must be to be considered as analogous (S1 Fig). In principle, use of a climate bin width as broad as the climatic niche width of the average species could reduce climatic velocity values to a level similar to biotic velocity. In practice, results from such an analysis would be uninformative due to the presence of large regions with zero velocity (S1 Fig).

Climatic velocity is often seen as a useful surrogate metric for assessment of climate exposure when species-specific information is lacking [1]. For example, climatic velocity may be used to assess vulnerability of localized endemic species which have too few observations with which to successfully construct niche models. However, our results suggest that biotic and climatic velocity can serve complementary roles within a conservation planning process even when species-specific information is available. Consideration of climatic velocity can help address conservation of locally-adapted populations, a facet of the extinction crisis that is often ignored in conservation planning [45]. Comparison of the geographic patterns of biotic and climatic velocity also helps planners distinguish regions where climatic velocity may be an adequate surrogate for climatic threats to biodiversity from regions where biogeographic factors play a greater role in influencing species vulnerability.

### Strengths and limitations of analog-based velocity vs. local velocity

Previous reviews have emphasized the importance of considering fine-scale topography that may create microrefugia that allow species to persist in place, as well as the large-scale climate patterns that can push species off mountaintops or the poleward edges of continents [5]. Because our method for calculating analog-based velocity is computationally efficient for even high-resolution climate surfaces, it can capture both fine and broad-scale factors within a single metric. However, analog-based velocity does carry some limitations compared to local velocity (Fig 1), and thus is best viewed as a complement to the latter metric, especially for vagile species which can disperse beyond the extent of a local neighborhood.

Recent studies have used local climatic velocity to estimate the movement vectors or paths that would allow organisms to track climatic conditions over broader continental scales [6]. Such broader-scale context is important for identifying geographic barriers to connectivity under climate change. The nearest-neighbor search method used to develop analog-based climatic velocity does allow sources, destinations, and directional vectors to be identified [7]. However, in the analog-based method, identification of the actual paths on the landscape that optimally connect sources and destinations requires additional analysis, for example using corridor delineation software [46]. Such connectivity analyses may incorporate barriers due to human land use as well as information on climatic gradients [25, 47].

Climatic velocity values depend to some degree on user choices that include the number of climate variables considered, the spatial resolution of the data, and the parameter which defines what degree of similarity constitutes a climate analog [7, 48]. However, results have been found to be robust in terms of the spatial arrangements of velocity values and thus the relative threat levels assigned to different regions [7].

### Geographic patterns of threat

Our hypothesis that future projected climatic velocity would peak in boreal regions was not supported. Our results instead suggest that areas of high future climatic velocity will not be confined to areas such as the boreal region which have experienced high climatic velocity in the past, but will extend into equatorial regions which have previously experienced low climatic velocity [14]. Previous studies have proposed that the narrower thermal tolerances of tropical organisms make them most vulnerable to climate change despite a lower absolute magnitude of climate change in tropical regions [49]. In our results, however, threats to equatorial species arise from both the future magnitude of exposure to climatic change (climatic velocity) and the narrow climatic tolerances of species in these equatorial regions, which currently experience low seasonal and interannual climatic variability [50–52].

This result agrees with previous studies which found that tropical regions, and the Amazon basin in particular, were highly threatened by future climate change. Projected future change in monthly temperature, when expressed in standard deviations from historical values rather than as absolute temperature change, projects greatest change to occur in tropical regions [53]. The Amazon basin has been ranked among the global regions with the highest distances to areas with projected future temperatures similar or cooler than current temperatures [54], and also among the regions most dominated by future novel climates [23, 55]. Although the equatorial zone generally showed higher exposure than other latitudes, a wide range of velocities was also evident at finer scales within latitudinal zones (Fig 3 and S4 Fig), illustrating the importance of identifying and protecting refugia within otherwise vulnerable areas.

Plotting the hemisphere’s protected areas in terms of the four velocity metrics (Fig 6) reveals that most equatorial protected areas are highly vulnerable from the perspective of both forward and backward biotic velocities as well as forward climatic velocity. In contrast, polar protected areas showed a wide range of vulnerability to both climatic velocity and forward biotic velocity, and thus appropriate adaptation strategies will differ more widely in this region. Similar quadrant plots of forward and backward biotic and climatic velocity can be applied at a finer scale to inform regional or local planning efforts.

The species distributions used to develop biotic velocity estimates may be limited by historical biogeographic factors and limited dispersal ability, and thus underrepresent species’ potential climatic tolerances [56, 57]. However, this potential resilience is counterbalanced by the smaller population sizes and lower dispersal ability typical of narrow range species, which may place them at “double jeopardy” from climate change [56].

Previously, areas in Europe holding many restricted-range plant and butterfly species were found to hold climates that were more likely to be lost in the future [58]. However, our results suggest a low correlation between areas of disappearing climates and species in the Americas. This contrast may be due to the contrasting historical biogeography of Europe and the Americas, or the contrasting taxonomic focus of the two studies.

Biotic velocity is similar to climatic velocity in that it synthesizes a large volume of data into a single metric that facilitates comprehension of common patterns across a range of variables. The contrasts in vulnerability between climate zones we observed appear robust in that they are not driven by a few highly vulnerable species (S6 Fig). Nonetheless, there is substantial variation in the velocities experienced by different species within the large taxonomic groups we considered (S6 Fig). Although aggregate metrics are a useful tool for discerning broad-scale threat patterns, conservation strategies will ultimately require consideration of individualistic species responses.

## Conclusions

Conservation planners have long realized that any one metric or set of targets can serve as only an imperfect surrogate for the larger goal of biodiversity conservation [59]. Our results suggest that geographic patterns of vulnerability to climate change differ depending on whether conservation of locally-adapted populations, species, or sites is of primary interest. This contrast should be considered when identifying areas which may function as refugia or conversely are likely to be at high risk of biodiversity loss under climate change.

Recent reviews have advocated consideration of multiple metrics of exposure to climate change [1]. One widely-adopted approach integrates representation of coarse-filter or non-species-specific information with fine-filter information on individual species [60]. Consideration of both climatic and biotic velocity allows such a coarse-filter/fine-filter approach to be extended to address the novel context of conservation under climate change [61].

## Supporting Information

S1 Fig Results of analysis of sensitivity of climatic velocity to width of bin used to define “matching” climate types. Sensitivity is defined in terms of a) median distance to climate match, and b) proportion of the resulting map composed of no-analog climates.
(PDF)

S2 Fig Variation in velocity values (standard deviation, in km/year) between species within a taxa group (birds, mammals, amphibians), and between mean values for the ten GCM projections. (PDF)

S3 Fig Distribution of values for climatic (a) and biotic (b-d) velocity, grouped by major climatic zones, based on kernel density estimation of the probability distribution function. Climatic zones are as shown in Fig 6.
(PDF)

S4 Fig Distribution of values for forward (top row) and backward (bottom row) climatic and biotic velocity, grouped by latitudinal band. Values for biotic velocity were first averaged over all species for each cell, and then the spatial variation in per-cell mean values across each latitudinal band was depicted using a beanplot. Solid black lines show mean values.
(PDF)

S5 Fig Spearman rank correlations between climatic velocity and biotic velocity for three taxa (birds, mammals, and amphibians), for the five major Koppen-Geiger climatic zones of the Americas (equatorial (E), arid (A), temperate (T), cold (C), and polar (P)). Upper case and lower case letters represent forward and backward velocity, respectively. Amphibian species were largely absent from polar regions.
(PDF)

S6 Fig Distribution of values within taxa groups of mean forward biotic velocity for individual species. Values for biotic velocity were first averaged over each climatic zone on a per-species basis and then between-species variation was depicted using a beanplot. Solid black lines show mean values.
(PDF)

S1 File R scripts for calculating analog-based biotic and climatic velocity. (ZIP)

S1 Table General circulation models (GCM) used by Lawler et al. (2009) for projections of bioclimatic variables. (PDF)

S2 Table Bioclimatic variables used by Lawler et al. (2009). (PDF)
