# Correction: Biotic and Climatic Velocity Identify Contrasting Areas of Vulnerability to Climate Change

**DOI:** 10.1371/journal.pone.0142024

**Published:** 2015-10-29

**Authors:** PLOS ONE Staff

There are errors in Fig 2 of the published article. Please view the correct Fig 2 and its caption here. The publisher apologizes for the error.

**Fig 2:**
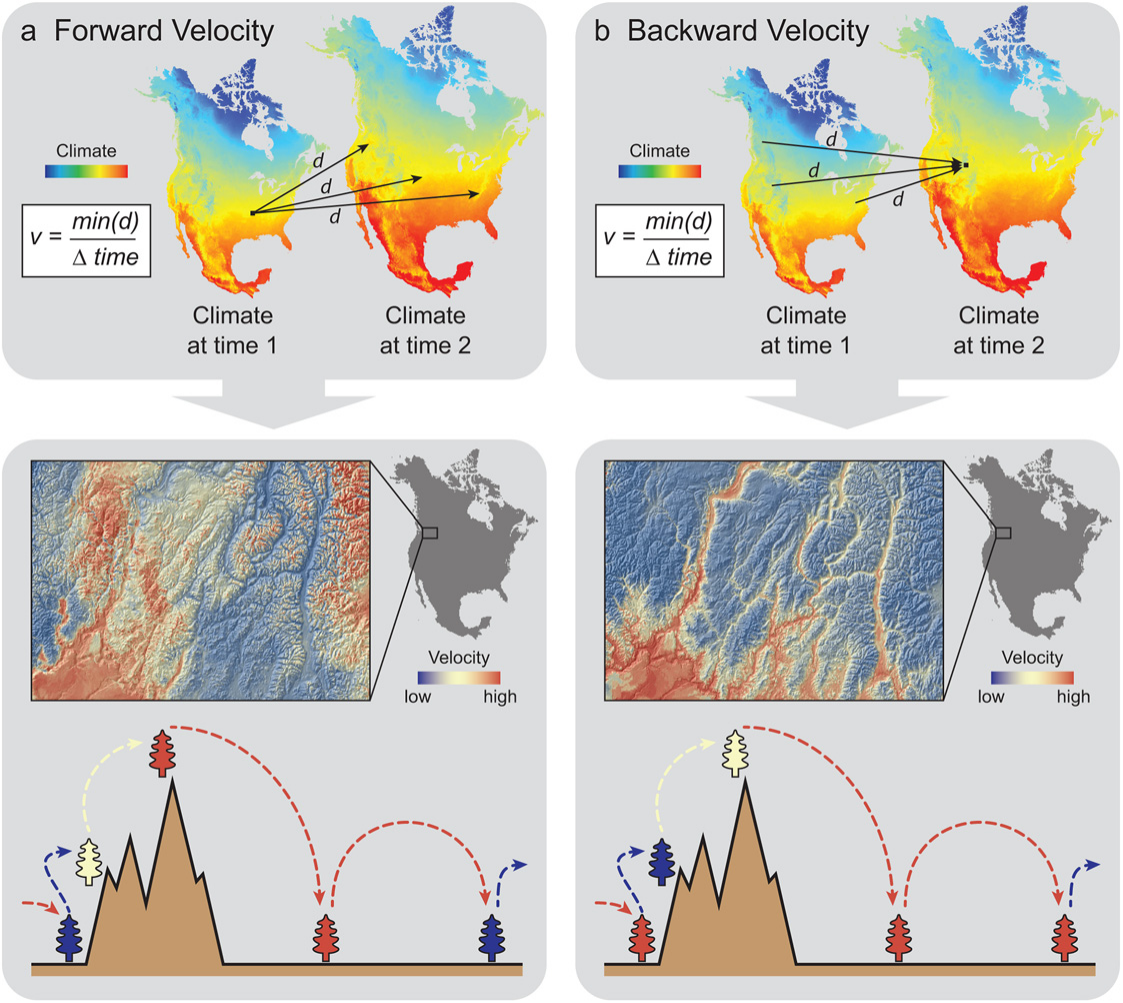
Conceptual diagram contrasting a) forward and b) backward velocity. Upper panels illustrate contrasts between how the two metrics are derived. Lower panels show a high resolution map of the contrast between forward and backward velocity in mountainous terrain, as well as a diagram illustrating the source of these contrasts. The colors of trees in the diagram correspond to the velocity expected in that topographic position, with the corresponding arrows of the same color either originating from (forward velocity) or terminating at that topographic position.
