# Supplementary material for: Biotic and Climatic Velocity Identify Contrasting Areas of Vulnerability to Climate Change
Source: PLoS One. 2015 Oct 14;10(10):e0140486. doi: 10.1371/journal.pone.0140486 (PMC4605713; doi:10.1371/journal.pone.0140486)
Supplement: S1 Fig — Sensitivity is defined in terms of a) median distance to climate match, and b) proportion of the resulting map composed of no-analog climates. (PDF) [file pone.0140486.s001.pdf]

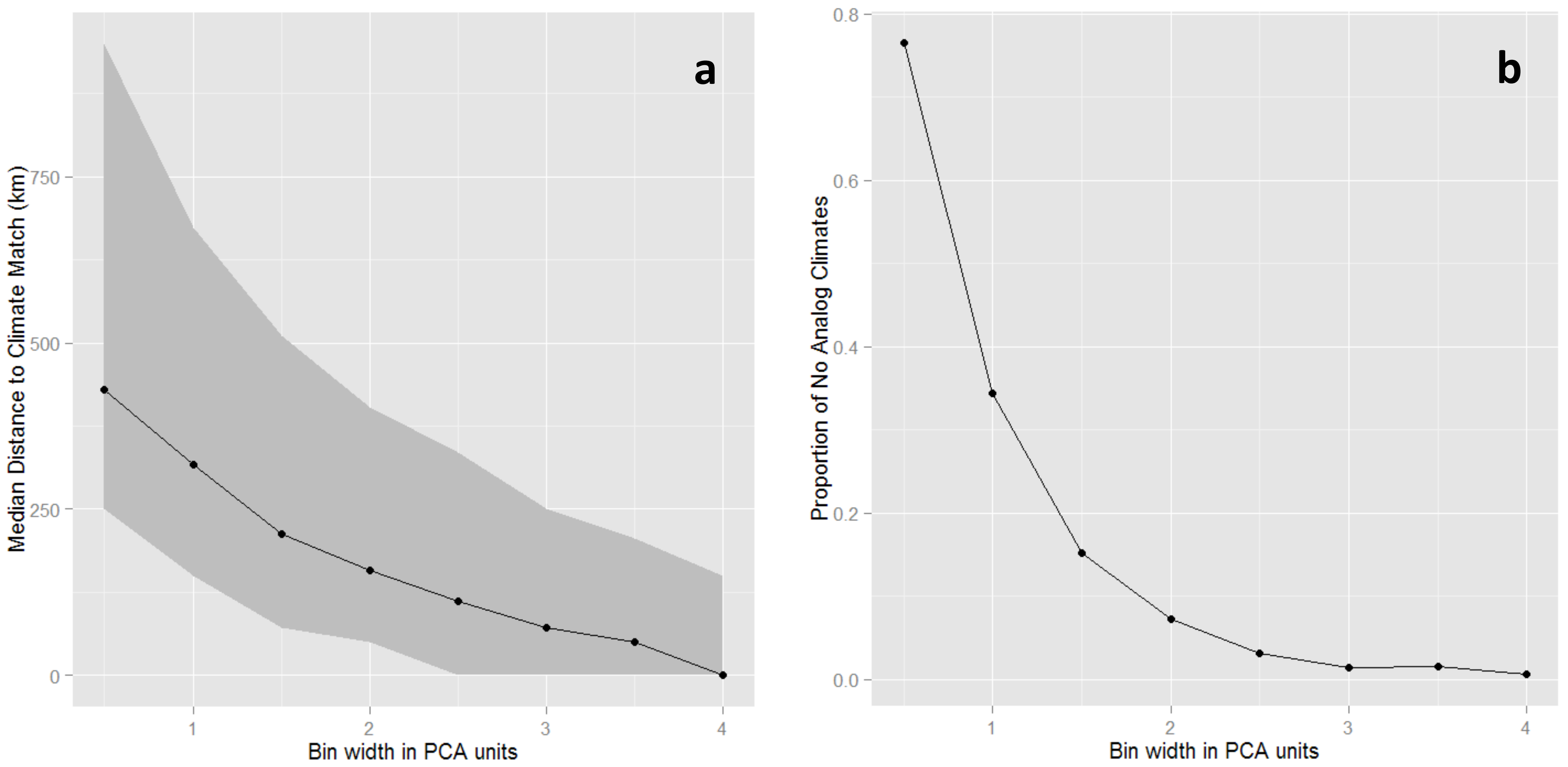

Figure S1. Results of analysis of sensitivity of climatic velocity to width of bin used to define “matching” climate types. Sensitivity is defined in terms of a) median distance to climate match, and b) proportion of the resulting map composed of no-analog climates. Gray band in a) shows 25% and 75% quantiles.

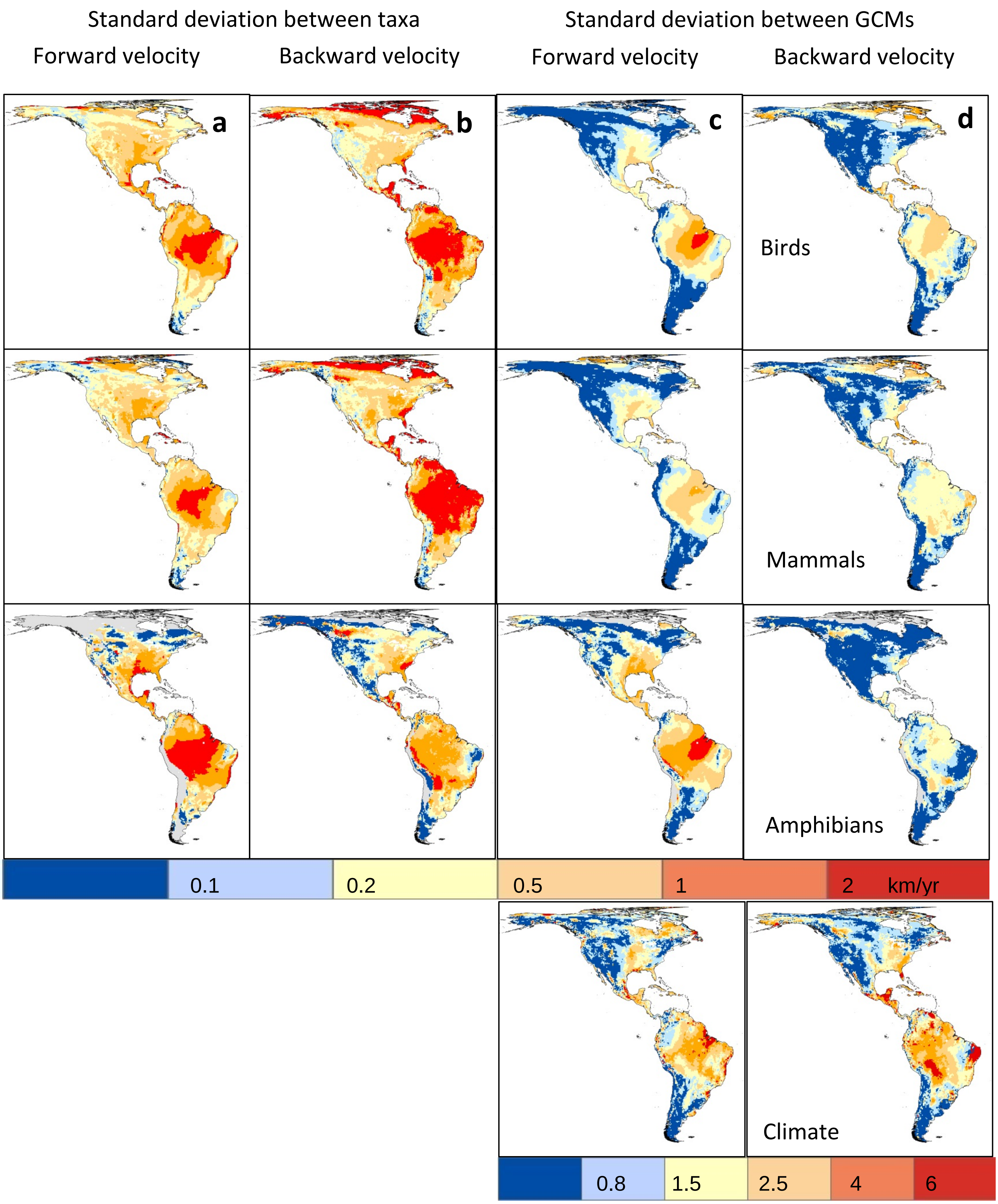

Figure S2. Variation in velocity values (standard deviation, in km/yr) between species within a taxa group (birds, mammals, amphibians), and between mean values for the 10 GCM projections.

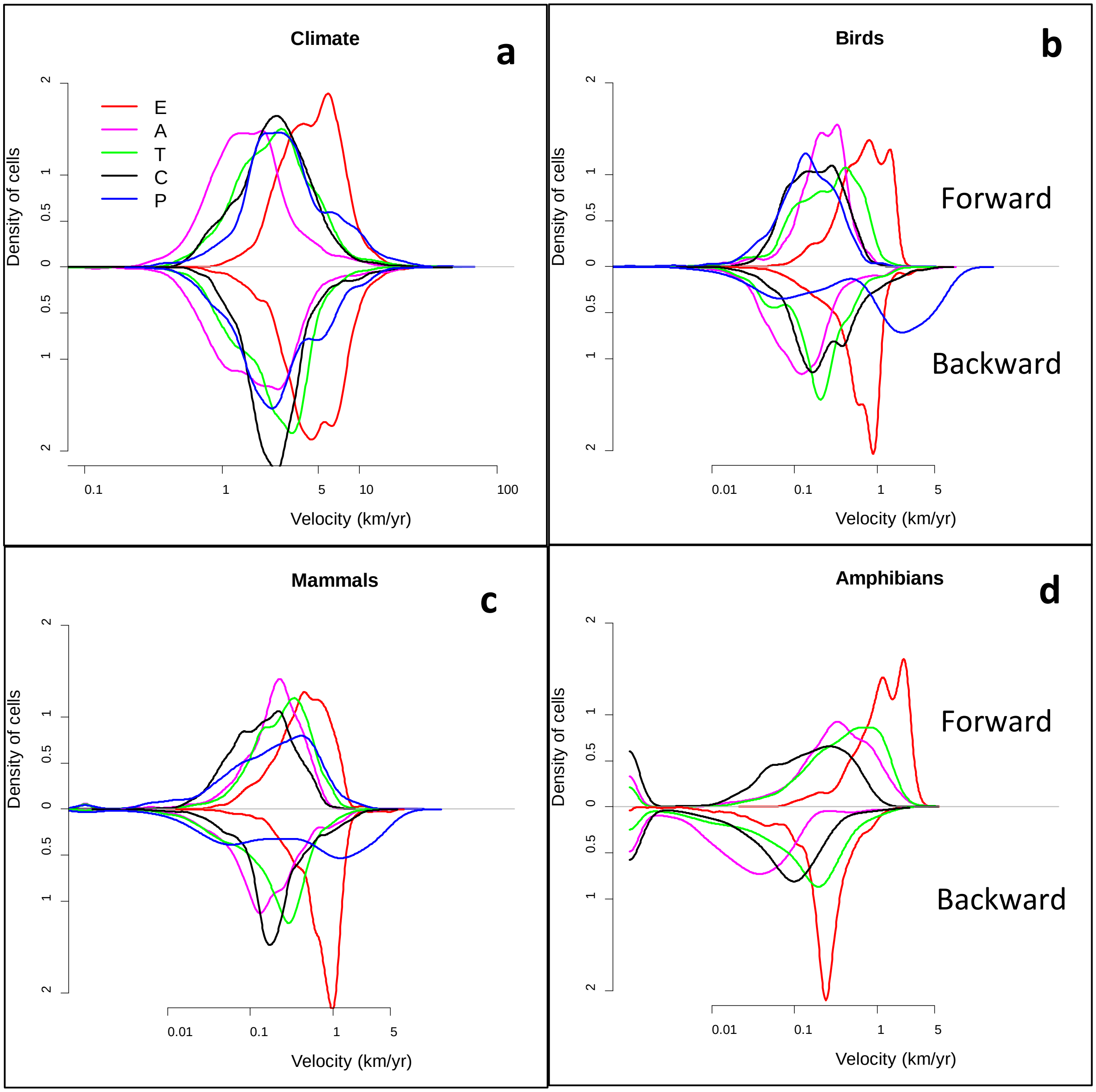

Figure S3. Distribution of values for climatic (a) and biotic (b-d) velocity, grouped by major climatic zones, based on kernel density estimation of the probability distribution function. Climatic zones are as shown in Fig. 6.

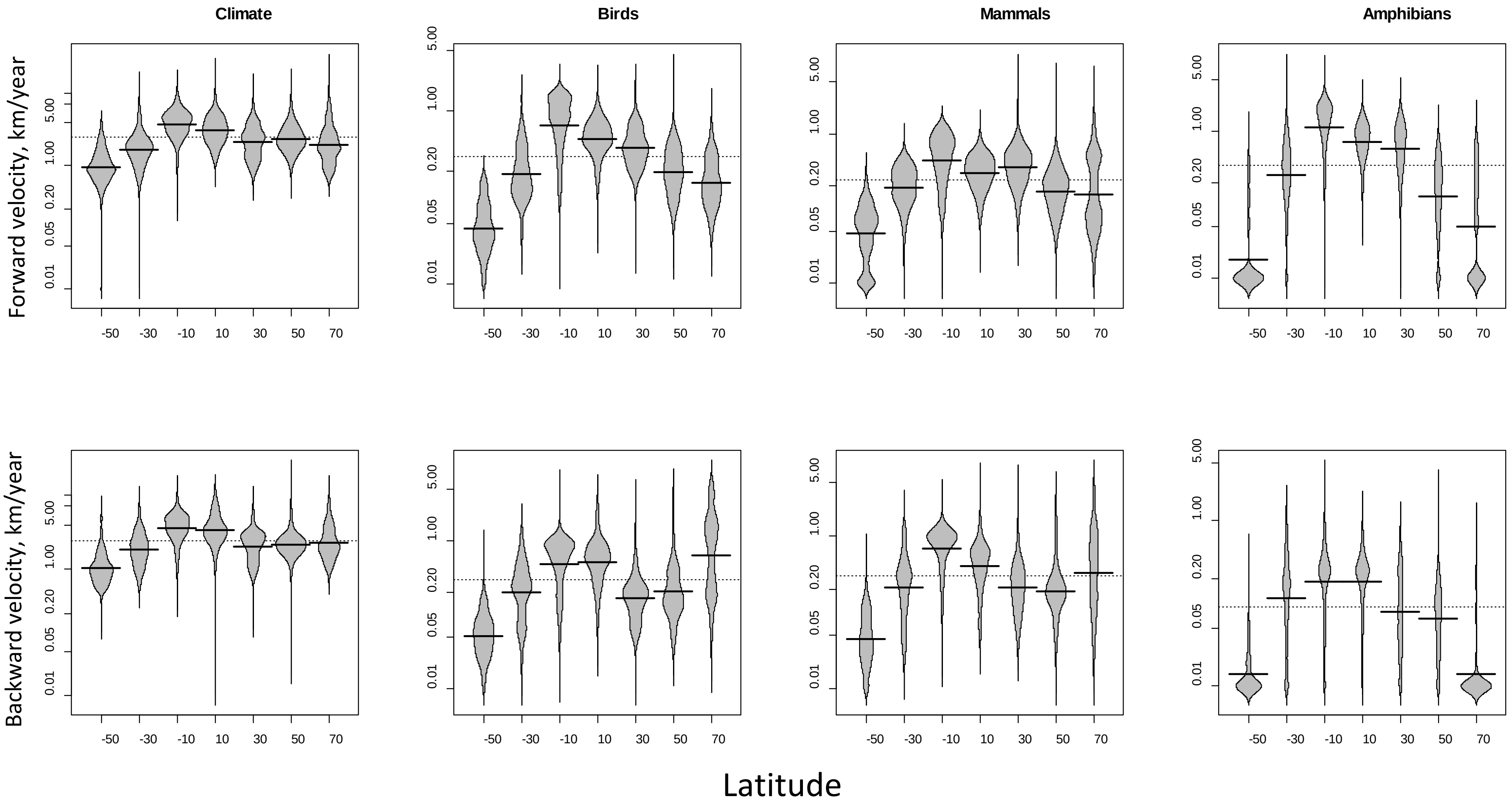

Figure S4. Distribution of values for forward (top row) and backward (bottom row) climatic and biotic velocity, grouped by latitudinal band. Values for biotic velocity were first averaged over all species for each cell, and then the spatial variation in per-cell mean values across each latitudinal band was depicted using a beanplot. Solid black lines show mean values.

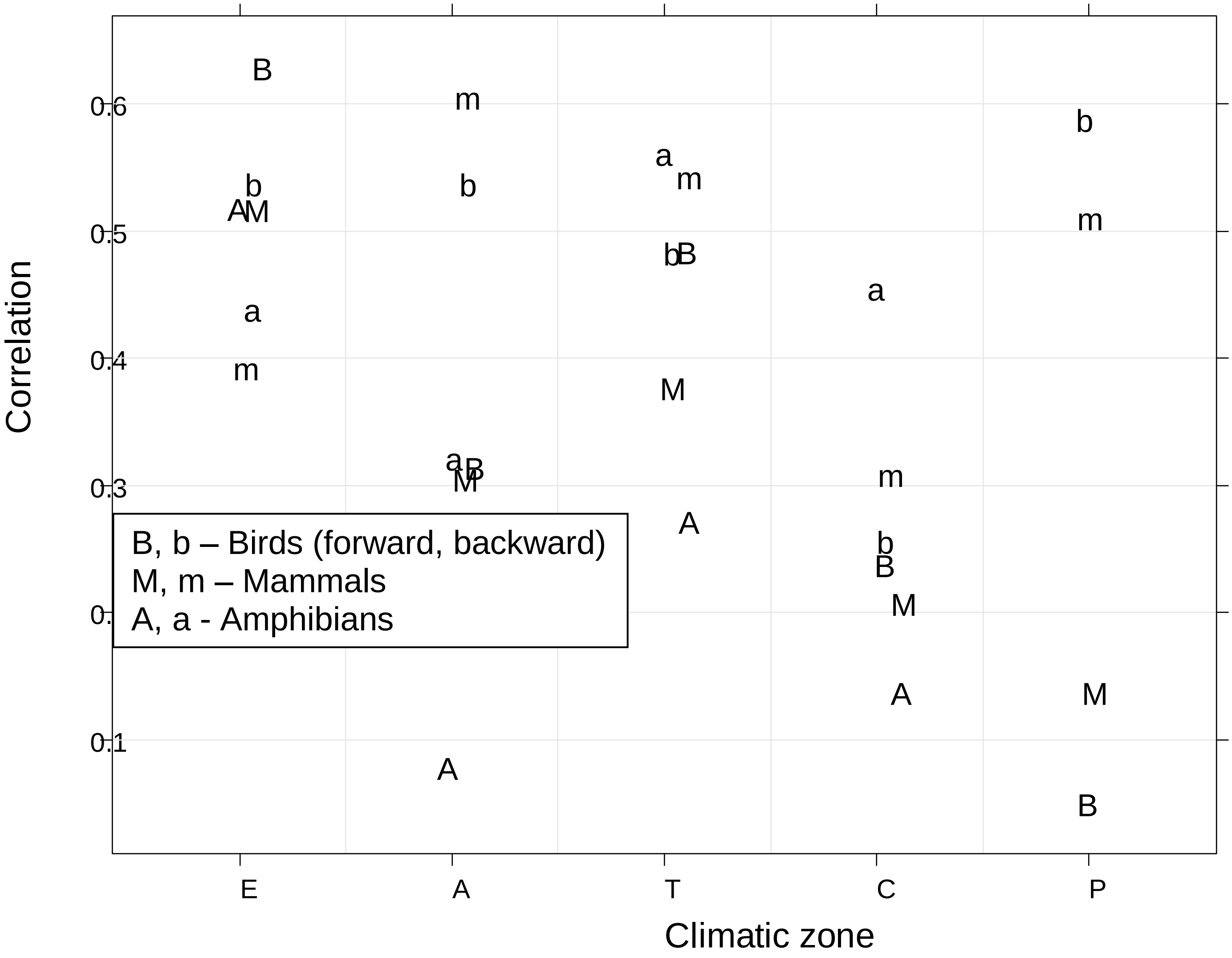

Figure S5. Spearman rank correlations between climatic velocity and biotic velocity for three taxa (birds, mammals, and amphibians), for the five major Koppen-Geiger climatic zones of the Americas (equatorial (E), arid (A), temperate (T), cold (C), and polar (P)). Upper case and lower case letters represent forward and backward velocity, respectively. Amphibian species were largely absent from polar regions.

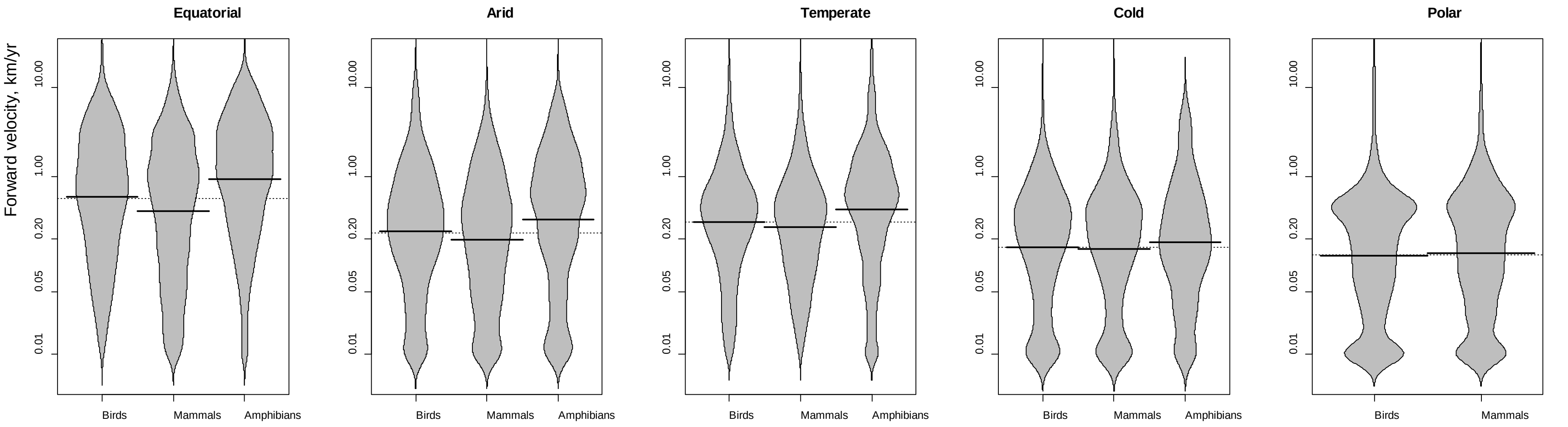

Figure S6. Distribution of values within taxa groups of mean forward biotic velocity for individual species. Values for biotic velocity were first averaged over each climatic zone on a per-species basis and then between-species variation was depicted using a beanplot. Solid black lines show mean values.

Table S1. General circulation models (GCM) used by Lawler et al. (2009) for projections of bioclimatic variables.

Model Name	Year	References
UKMO-HadCM3	1997	(Gordon et al. 2000, Pope et al. 2000)
MRI-CGCM2.3.2	2003	(Shibata et al. 1999, Yukimoto and Noda 2003)
CNRM-CM3	2004	(Déqué et al. 1994, Terray et al. 1998)
GISS-ER	2004	(Schmidt et al. 2006)
INM-CM3.0	2004	(Diansky and Volodin 2002, Galin et al. 2003)
MIROC3.2(medres)	2004	(K-1 Developers 2004)
CCSM3	2005	(Collins et al. 2006a, Collins et al. 2006b)
CGCM3.1(T47)	2005	(McFarlane et al. 1992, Flato 2005)
GFDL-CM2.0	2005	(Delworth et al. 2006)
GFDL-CM2.1	2005	(Delworth et al. 2006)

<u>References for Table S1.</u>

Collins, W. D., C. M. Bitz, M. L. Blackmon, et al. 2006*a*. The community climate system model version 3 (CCSM3). Journal of Climate 19:2122–2143.

Collins, W. D., P. J. Rasch, B. A. Boville, et al. 2006*b*. The formulation and atmospheric simulation of the Community Atmosphere Model Version 3 (CAM3). Journal of Climate 19:2144–2161.

Delworth, T. L., A. J. Broccoli, A. Rosati, et al. 2006. GFDL's CM2 global coupled climate models Part 1: Formulation and simulation characteristics. Journal of Climate 19:643–674.

Diansky, N. A., and E. M. Volodin. 2002. Simulation of the present-day climate with a coupled atmosphere-ocean general circulation model. Izvestia, Atmospheric and Oceanic Physics 38:732–747.

Déqué, M., C. Dreveton, A. Braun, and D. Cariolle. 1994. The ARPEGE/IFS atmosphere model: A contribution to the French community climate modeling. Climate Dynamics 10:249–266.

Flato, G. M. 2005. The Third Generation Coupled Global Climate Model (CGCM3) (and included links to the description of the AGCM3 atmospheric model). http://www.cccma.bc.ec.gc.ca/models/cgcm2.shtml. Last viewed.

Galin, V. Y., E. M. Volodin, and S. P. Smyshliaev. 2003. Atmospheric general circulation model of INM RAS with ozone dynamics. Russian Meteorology and Hydrology 5:13-22.

Gordon, C., C. Cooper, C. A. Senior, et al. 2000. The simulation of SST, sea ice extents and ocean heat transports in a version of the Hadley Centre coupled model without flux adjustments. Climate Dynamics 16:147–168.

K-1 Developers. 2004. K-1 coupled model (MIROC) description. K-1 Technical Report 1. Center for Climate System Research, University of Tokyo, Tokyo, Japan.

McFarlane, N. A., G. J. Boer, J.-P. Blanchet, and M. Lazare. 1992. The Canadian Climate Centre second-generation general circulation model and its equilibrium climate. Journal of Climate 5:1013–1044.

Pope, V. D., M. L. Gallani, P. R. Rowntree, and R. A. Stratton. 2000. The impact of new physical parametrizations in the Hadley Centre climate model -- HadAM3. Climate Dynamics 16:123–146.

Schmidt, G. A., R. Ruedy, J. E. Hansen, et al. 2006. Present day atmospheric simulations using GISS ModelE: Comparison to in-situ, satellite and reanalysis data. Journal of Climate 19:153–192.

Shibata, K., H. Yoshimura, M. Ohizumi, M. Hosaka, and M. Sugi. 1999. A simulation of troposphere, stratosphere and mesosphere with an MRI/JMA98 GCM. Papers in Meteorology and Geophysics 50:15–53.

Terray, L., S. Valcke, and A. Piacentini. 1998. OASIS 2.2 Guide and Reference Manual. TR/CMGC/98-05, CERFACS, Toulouse, France.

Yukimoto, S., and A. Noda. 2003. Improvements of the Meteorological Research Institute global ocean-atmosphere coupled GCM (MRI-GCM2) and its climate sensitivity. CGER's Supercomputing Activity Report. National Institute for Environmental Studies, Ibaraki, 305-0053 Japan.

Table S2. Bioclimatic variables used by Lawler et al. (2009).

1. Growing degree days (0 °C base)

2. Growing degree days (5 °C base)

3. Chilling period (number of days in the year with a mean temperature <= 5 °C)

4. Mean temperature of the coldest month (°C)

5. Mean temperature of the warmest month (°C)

6. Mean annual temperature (°C)

7. Annual actual evapotranspiration (mm)

8. Annual potential evapotranspiraton (mm)

9. Moisture index (annual actual evapotranspiration/annual potential evapotranspiration)

10. Actual evapotranspiration (mm) for days with temperatures > -4 °C

11. Potential evapotranspiration (mm) for days with temperatures > -4 °C

12. Moisture index for days with temperatures > -4 °C (annual actual evapotranspiration/annual potential evapotranspiration)

13. Actual evapotranspiration (mm) for days with temperatures > 5 °C

14. Potential evapotranspiration (mm) for days with temperatures > 5 °C

15. Moisture index for days with temperatures > 5 °C (annual actual evapotranspiration/annual potential evapotranspiration)

16. Total annual snow (mm)

17. March - May actual evapotranspiration (mm)

18. March - May potential evapotranspiration (mm)

19. March - May moisture index (actual evapotranspiration/potential evapotranspiration)

20. June - August actual evapotranspiration (mm)

21. June - August potential evapotranspiration (mm)

22. June - August moisture index (actual evapotranspiration/potential evapotranspiration)

23. September - November actual evapotranspiration (mm)

24. September - November potential evapotranspiration (mm)

25. September - November moisture index (actual evapotranspiration/potential evapotranspiration)

26. December - February actual evapotranspiration (mm)

27. December - February potential evapotranspiration (mm)

28. December - February moisture index (actual evapotranspiration/potential evapotranspiration)

29. March - May total precipitation (mm)

30. June - August total precipitation (mm)

31. September - November total precipitation (mm)

32. December - February total precipitation (mm)

33. Total annual precipitation (mm)

34. Mean monthly precipitation (mm) for the driest month

35. Mean monthly precipitation (mm) for the wettest month

36. Annual temperature range (warmest month minus coldest month)

37. Annual precipitation range (wettest month minus driest month)
