# Supplementary material for: Biotic and Climatic Velocity Identify Contrasting Areas of Vulnerability to Climate Change
Source: PLoS One. 2015 Oct 14;10(10):e0140486. doi: 10.1371/journal.pone.0140486 (PMC4605713; doi:10.1371/journal.pone.0140486)
Supplement: S2 Fig — (PDF) [file pone.0140486.s002.pdf]

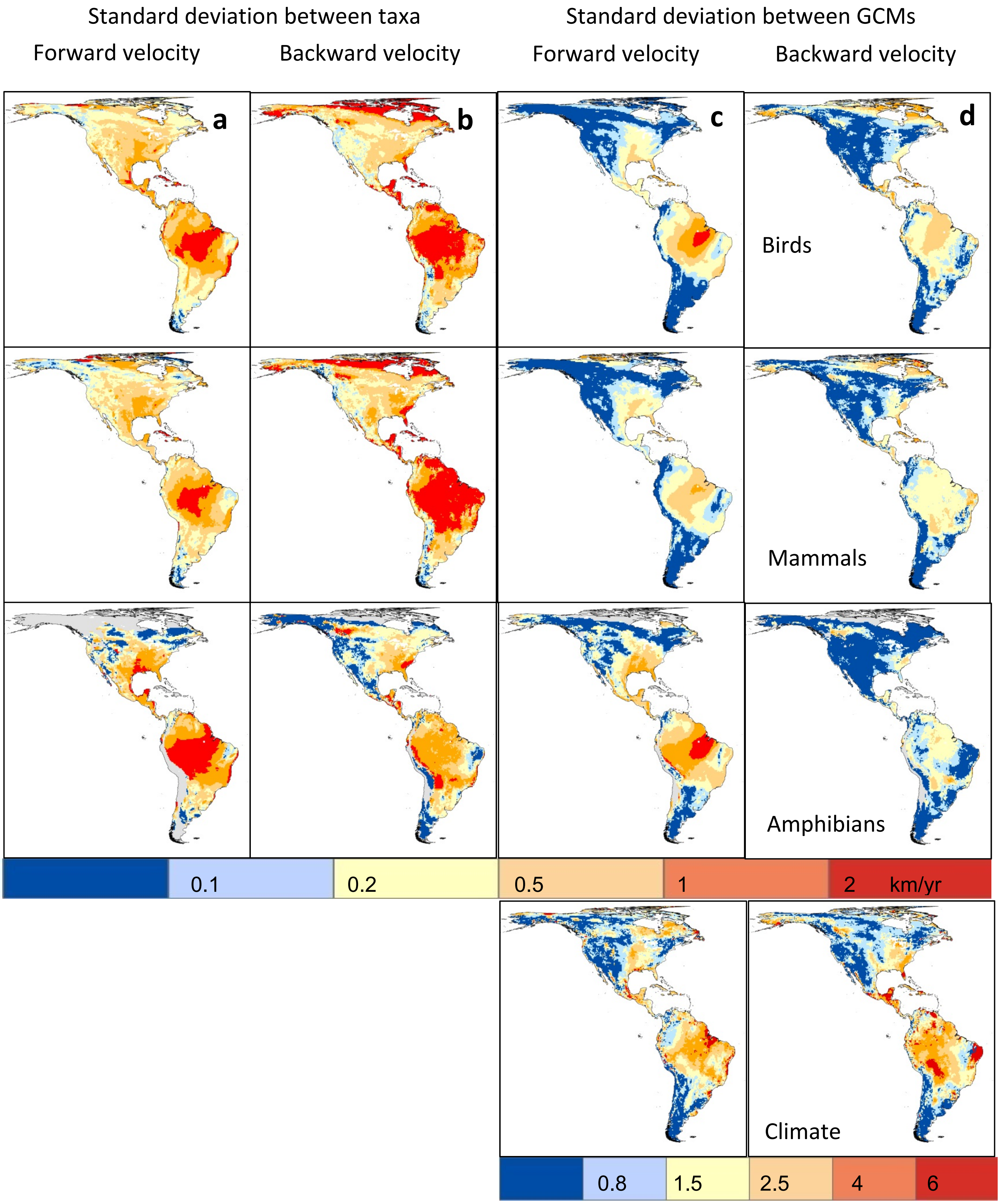

Figure S2. Variation in velocity values (standard deviation, in km/yr) between species within a taxa group (birds, mammals, amphibians), and between mean values for the 10 GCM projections.
