# Supplementary material for: Biotic and Climatic Velocity Identify Contrasting Areas of Vulnerability to Climate Change
Source: PLoS One. 2015 Oct 14;10(10):e0140486. doi: 10.1371/journal.pone.0140486 (PMC4605713; doi:10.1371/journal.pone.0140486)
Supplement: S3 Fig — Climatic zones are as shown in Fig 6. (PDF) [file pone.0140486.s003.pdf]

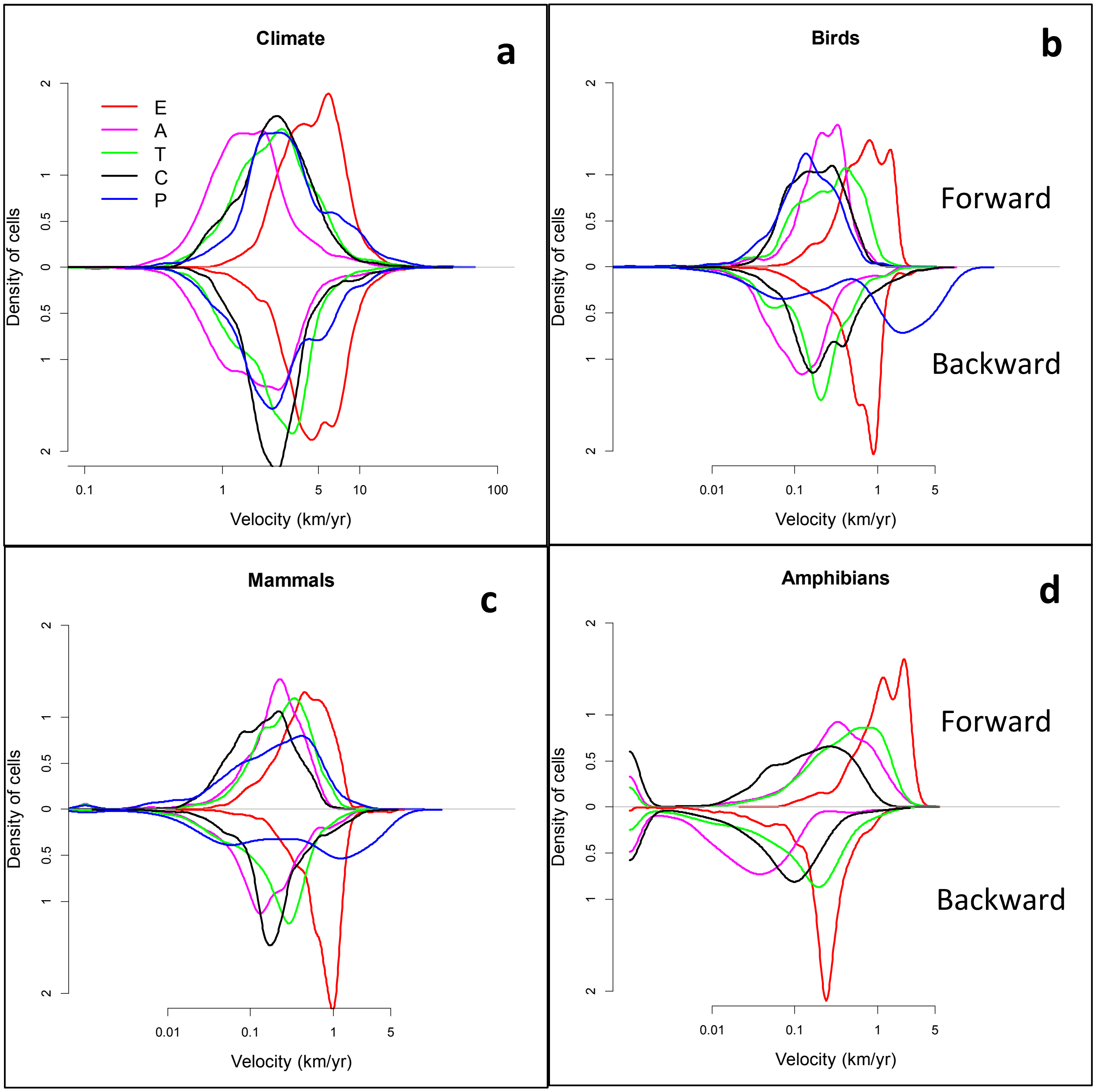

Figure S3. Distribution of values for climatic (a) and biotic (b-d) velocity, grouped by major climatic zones, based on kernel density estimation of the probability distribution function. Climatic zones are as shown in Fig. 6.
