# Supplementary material for: Biotic and Climatic Velocity Identify Contrasting Areas of Vulnerability to Climate Change
Source: PLoS One. 2015 Oct 14;10(10):e0140486. doi: 10.1371/journal.pone.0140486 (PMC4605713; doi:10.1371/journal.pone.0140486)
Supplement: S4 Fig — Values for biotic velocity were first averaged over all species for each cell, and then the spatial variation in per-cell mean values across each latitudinal band was depicted using a beanplot. Solid black lines show mean values. (PDF) [file pone.0140486.s004.pdf]

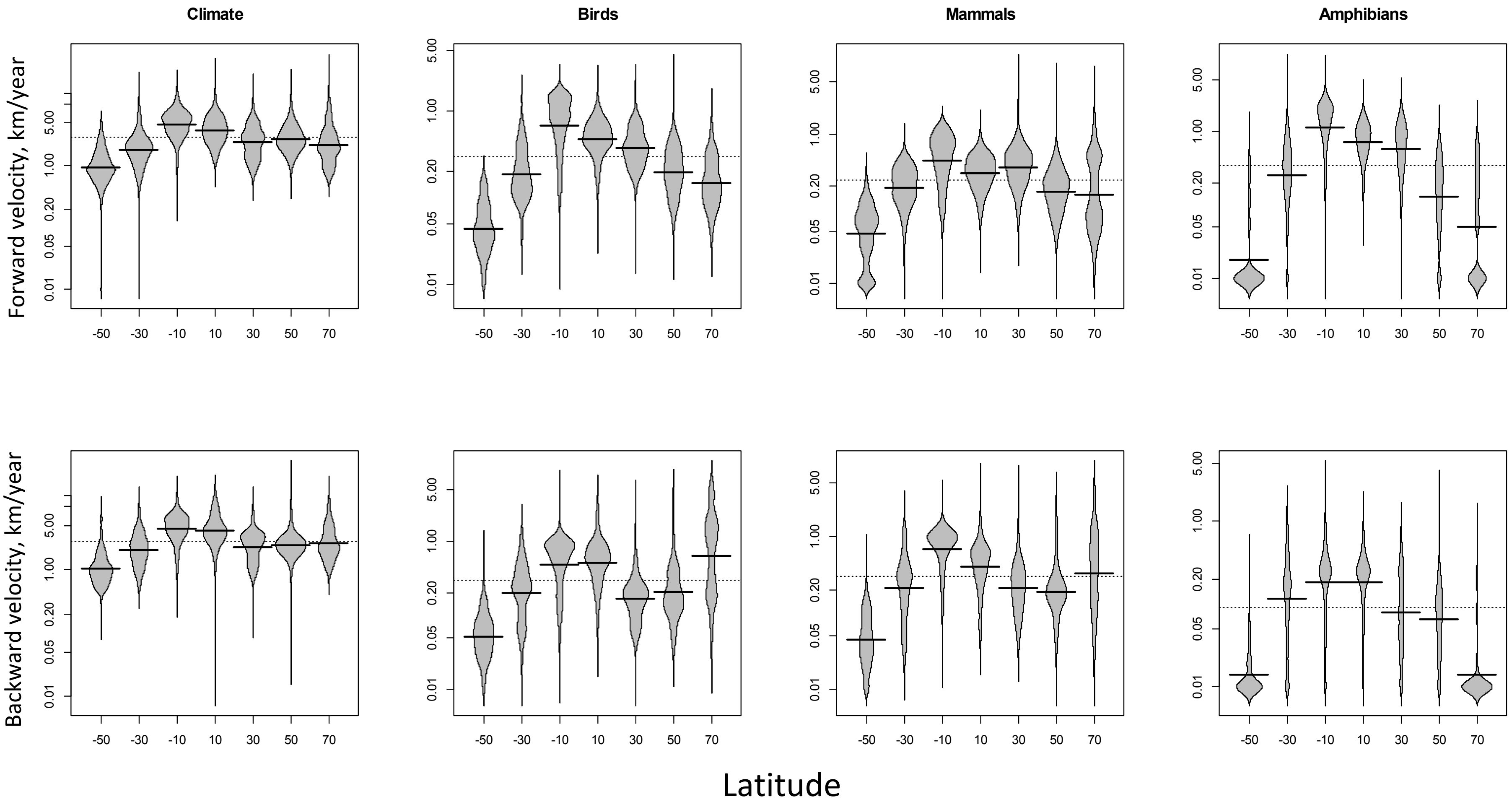

Figure S4. Distribution of values for forward (top row) and backward (bottom row) climatic and biotic velocity, grouped by latitudinal band. Values for biotic velocity were first averaged over all species for each cell, and then the spatial variation in per-cell mean values across each latitudinal band was depicted using a beanplot. Solid black lines show mean values.
