# Supplementary material for: Biotic and Climatic Velocity Identify Contrasting Areas of Vulnerability to Climate Change
Source: PLoS One. 2015 Oct 14;10(10):e0140486. doi: 10.1371/journal.pone.0140486 (PMC4605713; doi:10.1371/journal.pone.0140486)
Supplement: S5 Fig — Upper case and lower case letters represent forward and backward velocity, respectively. Amphibian species were largely absent from polar regions. (PDF) [file pone.0140486.s005.pdf]

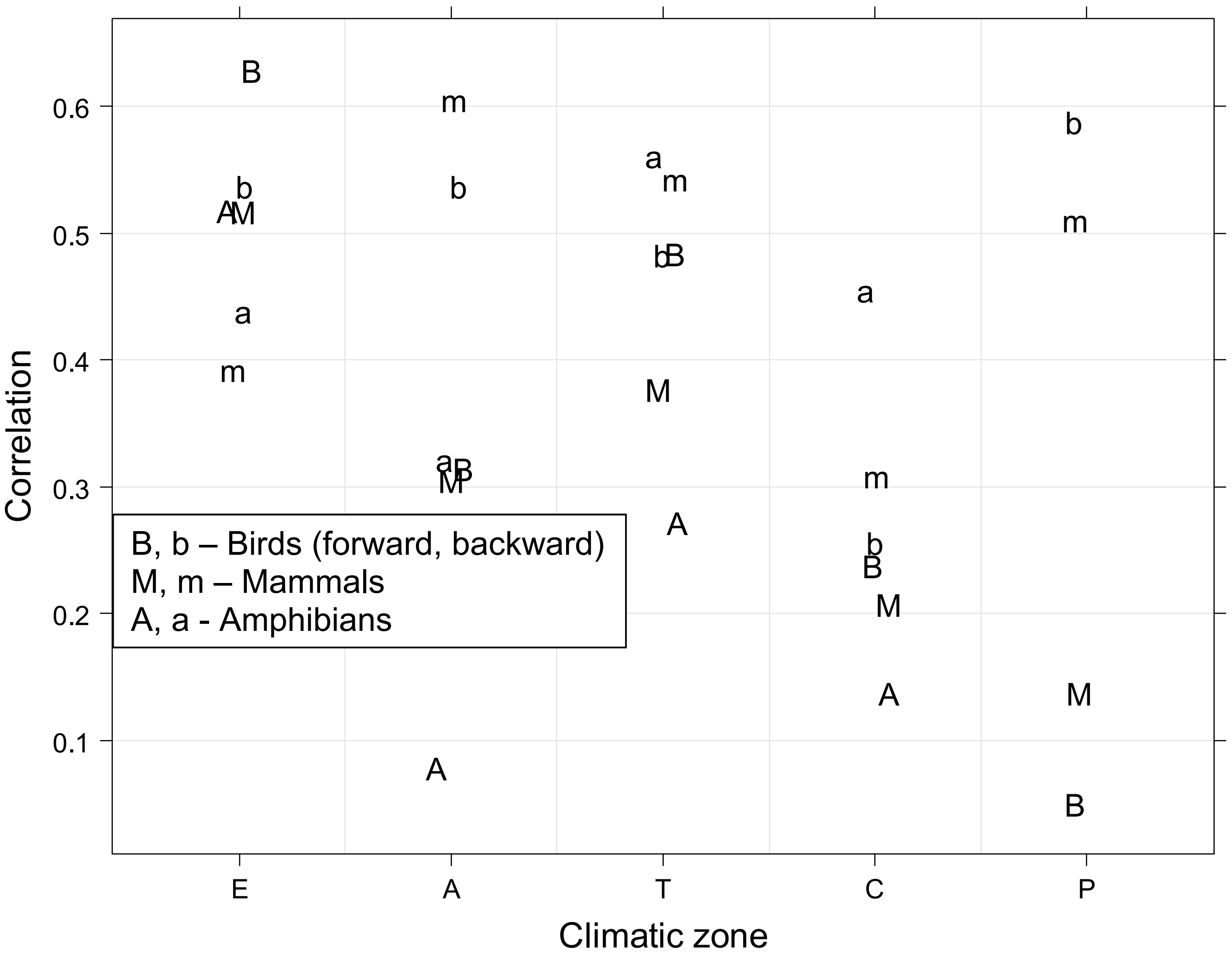

Figure S5. Spearman rank correlations between climatic velocity and biotic velocity for three taxa (birds, mammals, and amphibians), for the five major Koppen-Geiger climatic zones of the Americas (equatorial (E), arid (A), temperate (T), cold (C), and polar (P)). Upper case and lower case letters represent forward and backward velocity, respectively. Amphibian species were largely absent from polar regions.
