# Supplementary material for: Biotic and Climatic Velocity Identify Contrasting Areas of Vulnerability to Climate Change
Source: PLoS One. 2015 Oct 14;10(10):e0140486. doi: 10.1371/journal.pone.0140486 (PMC4605713; doi:10.1371/journal.pone.0140486)
Supplement: S6 Fig — Values for biotic velocity were first averaged over each climatic zone on a per-species basis and then between-species variation was depicted using a beanplot. Solid black lines show mean values. (PDF) [file pone.0140486.s006.pdf]

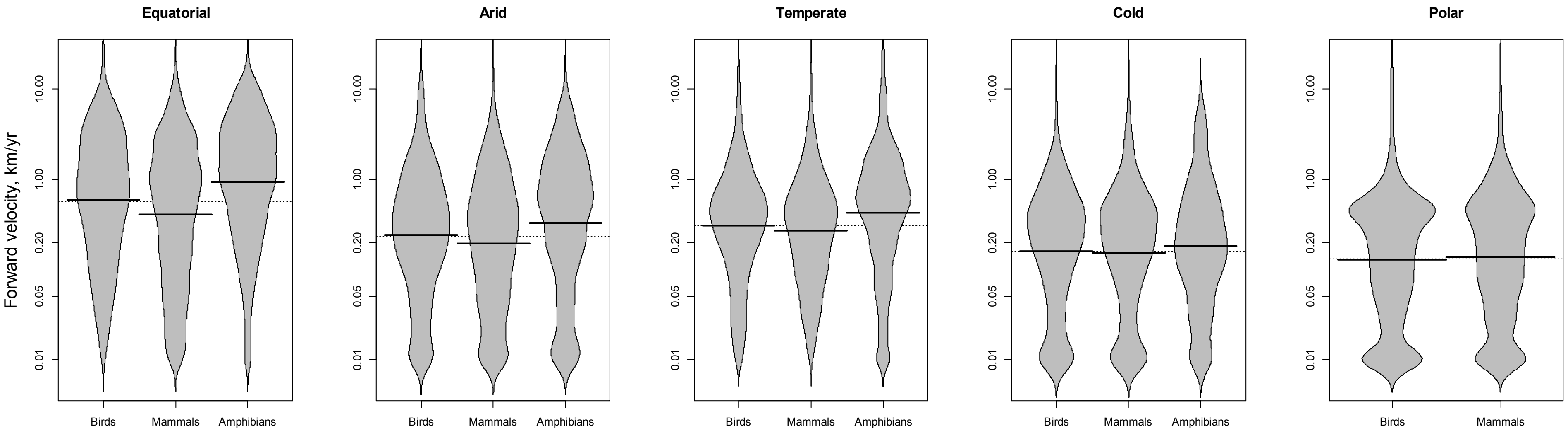

Figure S6. Distribution of values within taxa groups of mean forward biotic velocity for individual species. Values for biotic velocity were first averaged over each climatic zone on a per-species basis and then between-species variation was depicted using a beanplot. Solid black lines show mean values.
