# Supplementary material for: Biotic and Climatic Velocity Identify Contrasting Areas of Vulnerability to Climate Change
Source: PLoS One. 2015 Oct 14;10(10):e0140486. doi: 10.1371/journal.pone.0140486 (PMC4605713; doi:10.1371/journal.pone.0140486)
Supplement: S1 Table — (PDF) [file pone.0140486.s008.pdf]

Table S1. General circulation models (GCM) used by Lawler et al. (2009) for projections of bioclimatic variables.

Model Name	Year	References
UKMO-HadCM3	1997	(Gordon et al. 2000, Pope et al. 2000)
MRI-CGCM2.3.2	2003	(Shibata et al. 1999, Yukimoto and Noda 2003)
CNRM-CM3	2004	(Déqué et al. 1994, Terray et al. 1998)
GISS-ER	2004	(Schmidt et al. 2006)
INM-CM3.0	2004	(Diansky and Volodin 2002, Galin et al. 2003)
MIROC3.2(medres)	2004	(K-1 Developers 2004)
CCSM3	2005	(Collins et al. 2006a, Collins et al. 2006b)
CGCM3.1(T47)	2005	(McFarlane et al. 1992, Flato 2005)
GFDL-CM2.0	2005	(Delworth et al. 2006)
GFDL-CM2.1	2005	(Delworth et al. 2006)

<u>References for Table S1.</u>

Collins, W. D., C. M. Bitz, M. L. Blackmon, et al. 2006*a*. The community climate system model version 3 (CCSM3). Journal of Climate 19:2122–2143.

Collins, W. D., P. J. Rasch, B. A. Boville, et al. 2006*b*. The formulation and atmospheric simulation of the Community Atmosphere Model Version 3 (CAM3). Journal of Climate 19:2144–2161.

Delworth, T. L., A. J. Broccoli, A. Rosati, et al. 2006. GFDL's CM2 global coupled climate models Part 1: Formulation and simulation characteristics. Journal of Climate 19:643–674.

Diansky, N. A., and E. M. Volodin. 2002. Simulation of the present-day climate with a coupled atmosphere-ocean general circulation model. Izvestia, Atmospheric and Oceanic Physics 38:732–747.

Déqué, M., C. Dreveton, A. Braun, and D. Cariolle. 1994. The ARPEGE/IFS atmosphere model: A contribution to the French community climate modeling. Climate Dynamics 10:249–266.

Flato, G. M. 2005. The Third Generation Coupled Global Climate Model (CGCM3) (and included links to the description of the AGCM3 atmospheric model). http://www.cccma.bc.ec.gc.ca/models/cgcm2.shtml. Last viewed.

Galin, V. Y., E. M. Volodin, and S. P. Smyshliaev. 2003. Atmospheric general circulation model of INM RAS with ozone dynamics. Russian Meteorology and Hydrology 5:13-22.

Gordon, C., C. Cooper, C. A. Senior, et al. 2000. The simulation of SST, sea ice extents and ocean heat transports in a version of the Hadley Centre coupled model without flux adjustments. Climate Dynamics 16:147–168.

K-1 Developers. 2004. K-1 coupled model (MIROC) description. K-1 Technical Report 1. Center for Climate System Research, University of Tokyo, Tokyo, Japan.

McFarlane, N. A., G. J. Boer, J.-P. Blanchet, and M. Lazare. 1992. The Canadian Climate Centre second-generation general circulation model and its equilibrium climate. Journal of Climate 5:1013–1044.

Pope, V. D., M. L. Gallani, P. R. Rowntree, and R. A. Stratton. 2000. The impact of new physical parametrizations in the Hadley Centre climate model -- HadAM3. Climate Dynamics 16:123–146.

Schmidt, G. A., R. Ruedy, J. E. Hansen, et al. 2006. Present day atmospheric simulations using GISS ModelE: Comparison to in-situ, satellite and reanalysis data. Journal of Climate 19:153–192.

Shibata, K., H. Yoshimura, M. Ohizumi, M. Hosaka, and M. Sugi. 1999. A simulation of troposphere, stratosphere and mesosphere with an MRI/JMA98 GCM. Papers in Meteorology and Geophysics 50:15–53.

Terray, L., S. Valcke, and A. Piacentini. 1998. OASIS 2.2 Guide and Reference Manual. TR/CMGC/98-05, CERFACS, Toulouse, France.

Yukimoto, S., and A. Noda. 2003. Improvements of the Meteorological Research Institute global ocean-atmosphere coupled GCM (MRI-GCM2) and its climate sensitivity. CGER's Supercomputing Activity Report. National Institute for Environmental Studies, Ibaraki, 305-0053 Japan.
