# Supplementary material for: Biotic and Climatic Velocity Identify Contrasting Areas of Vulnerability to Climate Change
Source: PLoS One. 2015 Oct 14;10(10):e0140486. doi: 10.1371/journal.pone.0140486 (PMC4605713; doi:10.1371/journal.pone.0140486)
Supplement: S2 Table — (PDF) [file pone.0140486.s009.pdf]

Table S2. Bioclimatic variables used by Lawler et al. (2009).

1. Growing degree days (0 °C base)

2. Growing degree days (5 °C base)

3. Chilling period (number of days in the year with a mean temperature <= 5 °C)

4. Mean temperature of the coldest month (°C)

5. Mean temperature of the warmest month (°C)

6. Mean annual temperature (°C)

7. Annual actual evapotranspiration (mm)

8. Annual potential evapotranspiraton (mm)

9. Moisture index (annual actual evapotranspiration/annual potential evapotranspiration)

10. Actual evapotranspiration (mm) for days with temperatures > -4 °C

11. Potential evapotranspiration (mm) for days with temperatures > -4 °C

12. Moisture index for days with temperatures > -4 °C (annual actual evapotranspiration/annual potential evapotranspiration)

13. Actual evapotranspiration (mm) for days with temperatures > 5 °C

14. Potential evapotranspiration (mm) for days with temperatures > 5 °C

15. Moisture index for days with temperatures > 5 °C (annual actual evapotranspiration/annual potential evapotranspiration)

16. Total annual snow (mm)

17. March - May actual evapotranspiration (mm)

18. March - May potential evapotranspiration (mm)

19. March - May moisture index (actual evapotranspiration/potential evapotranspiration)

20. June - August actual evapotranspiration (mm)

21. June - August potential evapotranspiration (mm)

22. June - August moisture index (actual evapotranspiration/potential evapotranspiration)

23. September - November actual evapotranspiration (mm)

24. September - November potential evapotranspiration (mm)

25. September - November moisture index (actual evapotranspiration/potential evapotranspiration)

26. December - February actual evapotranspiration (mm)

27. December - February potential evapotranspiration (mm)

28. December - February moisture index (actual evapotranspiration/potential evapotranspiration)

29. March - May total precipitation (mm)

30. June - August total precipitation (mm)

31. September - November total precipitation (mm)

32. December - February total precipitation (mm)

33. Total annual precipitation (mm)

34. Mean monthly precipitation (mm) for the driest month

35. Mean monthly precipitation (mm) for the wettest month

36. Annual temperature range (warmest month minus coldest month)

37. Annual precipitation range (wettest month minus driest month)
